# Physical activity in relation to circulating hormone concentrations in 117,100 men in UK Biobank

**DOI:** 10.1007/s10552-021-01466-6

**Published:** 2021-07-03

**Authors:** Eleanor L. Watts, Aurora Perez-Cornago, Aiden Doherty, Naomi E. Allen, Georgina K. Fensom, Sandar Tin Tin, Timothy J. Key, Ruth C. Travis

**Affiliations:** 1 Cancer Epidemiology Unit, Nuffield Department of Population Health, University of Oxford, Richard Doll Building, Old Road Campus, Oxford, OX3 7LF UK; 2 Big Data Institute, Li Ka Shing Centre for Health Information and Discovery, University of Oxford, Oxford, UK; 3 Nuffield Department of Population Health, University of Oxford, Oxford, UK; 4 Clinical Trial Service Unit and Epidemiological Studies Unit, Nuffield Department of Population Health, University of Oxford, Oxford, UK; 5 UK Biobank Ltd, Cheadle, Stockport, UK; 6 National Institute of Health Research Oxford Biomedical Research Centre, Oxford, UK

**Keywords:** Physical activity, Testosterone, IGF-I, SHBG, Accelerometer, UK Biobank

## Abstract

**Purpose:**

Physical activity may reduce the risk of some types of cancer in men. Biological mechanisms may involve changes in hormone concentrations; however, this relationship is not well established. Therefore, we aimed to investigate the associations of physical activity with circulating insulin-like growth factor-I (IGF-I), sex hormone-binding globulin (SHBG, which modifies sex hormone activity), and total and free testosterone concentrations, and the extent these associations might be mediated by body mass index (BMI).

**Methods:**

Circulating concentrations of these hormones and anthropometric measurements and self-reported physical activity data were available for 117,100 healthy male UK Biobank participants at recruitment. Objectively measured accelerometer physical activity levels were also collected on average 5.7 years after recruitment in 28,000 men. Geometric means of hormone concentrations were estimated using multivariable-adjusted analysis of variance, with and without adjustment for BMI.

**Results:**

The associations between physical activity and hormones were modest and similar for objectively measured (accelerometer) and self-reported physical activity. Compared to men with the lowest objectively measured physical activity, men with high physical activity levels had 14% and 8% higher concentrations of SHBG and total testosterone, respectively, and these differences were attenuated to 6% and 3% following adjustment for BMI.

**Conclusion:**

Our results suggest that the associations of physical activity with the hormones investigated are, at most, modest; and following adjustment for BMI, the small associations with SHBG and total testosterone were largely attenuated. Therefore, it is unlikely that changes in these circulating hormones explain the associations of physical activity with risk of cancer either independently or via BMI.

**Supplementary Information:**

The online version contains supplementary material available at 10.1007/s10552-021-01466-6.

## Introduction

Higher physical activity levels are associated with a lower risk of some types of cancer in men [1]. Physical activity may be associated with cancer risk via changes in hormone concentrations, metabolism, immune function, body composition and/or by reducing low-grade inflammation [2–4]. However, clear biological mechanisms linking physical activity to cancer risk are not well established. There is consistent epidemiological evidence that the hormones insulin-like growth factor-I (IGF-I), sex hormone-binding globulin (SHBG: this molecule is not a hormone, but modifies sex hormone activity), and total and free testosterone may be related to cancer risk. In particular, higher IGF-I concentration has been associated with an increased risk of prostate, thyroid and colorectal cancers [5–8], while higher free testosterone has been associated with an increased risk of prostate cancer and possibly malignant melanoma in men [7, 9]. Previous studies investigating the possible effect of physical activity on serum hormone concentrations in men have been inconclusive [10–19]. However, observational studies were generally based on self-reported physical activity, with a limited sample size (up to 6,000) [10–13]. Randomised controlled trials generally have been of limited duration, potentially biased by participant dropout, and may have been underpowered to detect more modest associations [14–19]. Body mass index (BMI) also has well-established associations with IGF-I, SHBG, total and free testosterone concentrations [20, 21], and it is not clear whether, and to what extent, physical activity is associated with circulating hormone concentrations independently of BMI.

In this paper, we aimed to examine the associations of objectively measured and self-reported physical activity and sedentary time, as well as anthropometric factors, with serum concentrations of IGF-I, SHBG, and total and free testosterone in a cohort of 117,100 men; we recently reported similar analyses in women [22]. As adiposity may be important in the associations between physical activity and hormones, we first describe the associations of BMI with the hormones, and then the associations of physical activity with the hormones both without and with adjustment for BMI.

## Materials and methods

The design of the analyses, presentation and description are in accordance with the STROBE checklist [23].

### Study design

UK Biobank is a prospective cohort for public health research. Details of the study protocol and data collection are available online (http://www.ukbiobank.ac.uk/wp-content/uploads/2011/11/UK-Biobank-Protocol.pdf) and elsewhere [24, 25].

In brief, all participants were registered with the UK National Health Service and lived within 40 km of one of the UK Biobank assessment centres. Approximately 9.2 million people were initially invited to participate. Overall, 503,317 participants (229,122 men) aged 40–69 years consented to join the cohort and attended one of 22 assessment centres throughout England, Wales and Scotland between 2006 and 2010, a participation rate of 5.5% [25].

The UK Biobank study was approved by the North West Multi-Centre Research Ethics Committee (reference number 06/MRE08/65), and at recruitment, all participants gave written informed consent to participate and for their health to be followed-up through linkage to electronic medical records.

### Baseline assessment

At the baseline assessment visit, participants provided information on a range of sociodemographic, anthropometric, lifestyle, and health-related factors via a self-completed touch-screen questionnaire and a computer-assisted personal interview [25].

#### Anthropometry

Anthropometric measurements were taken at the assessment centre at study baseline [25]. Height was measured using the Seca 202 height measure (Seca, Hamburg, Germany), and waist and hip measurements were made over light clothes with the Seca‐200 tape measure (Seca, Hamburg, Germany). Weight and bio‐impedance measures were taken using the Tanita BC418MA body composition analyser (Tanita, Tokyo, Japan). BMI was calculated as weight (kg)/height (m)^2^. Comparative body size and height at age 10 were self-reported via the touch-screen questionnaire at recruitment.

#### Self-reported physical activity

Self‐reported physical activity was assessed using questions adapted from the International Physical Activity Questionnaire (IPAQ) short form, a validated survey based on the frequency and duration of walking, moderate and vigorous activity [26].

The duration of each level of activity was weighted by estimated metabolic equivalent of tasks (METs) (3.3, 4.0 and 8.0 METs for walking, moderate intensity and vigorous intensity, respectively), to estimate total MET hours per week of physical activity. Following IPAQ guidelines, physical activity for any category of less than 10 min per day was recoded to 0 and durations of > 180 min per day were truncated [26].

Sedentary time was estimated as the total hours per week that participants reported spending watching television or using a computer (not including work).

### Accelerometer assessment

Between February 2013 and December 2015, participants who provided a valid email address were selected at random to receive email invitations to wear an Axivity AX3 wrist-worn accelerometer for 7 days to objectively measure physical activity levels (44.8% response rate) [27]. Accelerometer-measured physical activity was defined as the vector magnitude of acceleration (in milligravity units) averaged over five‐second time windows [27]. This has been validated against the doubly labelled water method which is a gold standard measure for energy expenditure in participants with stable weight [28].

The percentages of time spent in moderate and vigorous, light tasks and walking physical activity as well as sedentary time measured by the accelerometer were calculated using machine learning methods that have been described in more detail elsewhere [29, 30]. These methods extracted time and frequency domain features from each 30 s window in the accelerometer time series. Random forests and hidden Markov models were then trained and evaluated in 153 free-living individuals (mean age = 42, male n = 53) to distinguish between activity states, evaluated against reference wearable camera, time-use and sleep diary information sources.

### Blood sampling and biomarker assays

At recruitment, blood sampling was successfully performed in 99.7% of the cohort. Blood was collected in a serum separator tube and shipped to the central processing laboratory in temperature-controlled boxes at 4 °C [31], then aliquoted and stored in a central working archive at − 80 °C [32]. Measurements of serum concentrations of IGF-I, SHBG, testosterone and albumin were attempted in all participants. IGF-I was determined by chemiluminescent immunoassays (DiaSorin Liaison XL), and SHBG and testosterone concentrations were measured using chemiluminescent immunoassays (Beckman Coulter Unicel DxI 800). Albumin was measured by a colorimetric assay (Beckman Coulter AU5800). Average within-laboratory (total) coefficients of variation for low, medium and high internal quality control level samples for each biomarker ranged from 2.1–8.3%. Full details of the assay methods and quality assurance protocols are available online (https://biobank.ndph.ox.ac.uk/showcase/docs/serum_biochemistry.pdf).

#### Free testosterone calculation

In the circulation, approximately 98% testosterone is bound to SHBG and albumin. The remaining 2% circulates as unbound or “free” testosterone and is hypothesised to be biologically active [33]. Free testosterone concentrations were estimated using a formula based on the law of mass action and measured total testosterone, SHBG and albumin concentrations [7, 34].

### Repeat measurements

Participants who lived within a 35-km radius were invited via email to attend a repeat assessment clinic at the UK Biobank Co-ordinating Centre in Stockport between August 2012 and June 2013. Repeat assessments including blood sampling and self-reported physical activity and sedentary behaviour were completed in 9,000 men with a response rate of 21% [35].

### Exclusion criteria

We excluded 9,869 men with prevalent cancer (except C44: non-melanoma skin cancer). We also excluded 13,524 men who did not have hormone measurements available or who had biomarker measurements that did not pass quality control procedures [36], 780 men who had no BMI data. Chronic illness and diabetes is associated with altered hormone concentrations [37] and also may affect engagement in physical activity; therefore, we excluded 15,074 men who reported having diabetes and 48,217 men who reported being in poor or fair overall health. We also excluded 1,226 participants for whom it was not possible to determine genetic sex or who were identified as being genetically female, and 1,290 men who reported taking hormone medication at baseline (Supplementary Figure S1).

For the analysis of accelerometer data, we also excluded 1,190 men who were diagnosed with cancer between baseline and accelerometer reading. We excluded 2,064 men with insufficient wear time, poor calibration, > 1% clipped values (which occur when the sensor’s dynamic range of + -8 g is exceeded before or after calibration), and participants with implausibly high activity values, as described elsewhere [27]. For analysis of self-reported physical activity data, we excluded 21,543 men with missing or incomplete physical activity data and 449 men who reported undertaking more than 16 h of physical activity per day [26].

In total, our analytical dataset included 117,143 healthy men with valid self-reported physical activity and 27,933 with accelerometer measurements (Supplementary Figure S1).

### Statistical analysis

IGF-I, SHBG and total and free testosterone concentrations were logarithmically transformed to approximate a normal distribution. Analyses examined associations with anthropometric factors, accelerometer-measured physical activity and self-reported physical activity. The anthropometric factors were BMI (< 22.5, ≥ 22.5–< 25, ≥ 25–< 27.5, ≥ 27.5–< 30.0, ≥ 30.0–< 35.0, ≥ 35 kg/m^2^), height (< 170, ≥ 170–< 175, ≥ 175–< 180, ≥ 180–< 185, ≥ 185 cm), waist circumference (cm, fifths), waist-to-hip ratio (WHR) (fifths), body size aged 10 (thinner, about average, plumper), height aged 10 (shorter, about average, taller). Measured physical activity variables were overall score (milligravity, fifths), moderate physical activity (% time spent, fifths), light tasks (% time spent, fifths), walking (% time spent, fifths) and sedentary (% time spent, fifths). Self-reported physical activity variables were total METs (hours per week, fifths), vigorous (hours per week, fifths), moderate (hours per week, fifths), walking (hours per week, fifths) and sedentary time (hours per week, fifths).

Geometric mean hormone concentrations were calculated using predicted values from analysis of variance (ANOVA) models scaled to the overall geometric mean concentration and adjusted for age at recruitment (< 45, ≥ 45–< 49, ≥ 50–< 54, ≥ 55–< 59, ≥ 60–< 64, ≥ 65 years), geographic area (10 UK regions), Townsend deprivation score (fifths, unknown (0.1%)), racial/ethnic group (white, mixed background, Asian, black, other and unknown (0.5%)), height (categories as above), cigarette smoking (never, former, current light smoker (1–< 15 cigarettes per day), current heavy smoker (≥ 15 cigarettes per day), current (number of cigarettes per day unknown), and smoking status unknown (0.6%)), and alcohol consumption (non-drinkers, < 1–< 10, ≥ 10–< 20, ≥ 20 g ethanol/day, unknown (0.5%)). In analyses of height as the exposure, height was not included as an adjustment factor and in analyses of physical activity. The primary model was also further adjusted for BMI (categories as above). Adjustment covariates were defined a priori based on previous analyses of UK Biobank data [22, 38], and categories were used to account for nonlinear associations.

Heterogeneity of means by category was tested using the F test. P_trend_ was estimated using the ANOVA test with the categorical variables entered as linear values scored consecutively as the median values within each quantile.

### Further analyses

To compare the magnitudes of the associations of anthropometric and physical activity measures with hormone concentrations, and the role of possible confounders in the associations, we estimated percentage change in hormone concentration per 1 SD increase using minimally adjusted (adjusted for age and region categories as defined above) and multivariable-adjusted ANOVA models with standardised continuous exposure variables.

Repeat hormone concentrations and accelerometer scores were available in a subset of up to 2,372 men, and repeat measures of both hormones and self-reported physical activity were available in up to 6,027 men. To assess the robustness of our results, analyses were repeated using (i) the single accelerometer score and mean baseline and repeat hormone measurements, and (ii) the means of baseline and repeat assessment values for both self-reported physical activity and hormone measurements. These associations were compared to the baseline only associations in this subset.

In further analyses, we tested for heterogeneity by the possible effect modifiers: (i) BMI category (< 25, ≥ 25–< 27.5, ≥ 27.5–< 30, ≥ 30 kg/m^2^), (ii) employment status (employed or self-employed vs not employed or self-employed), (iii) regularity of heavy manual or physical work (sometimes/usually/always vs never) using the likelihood ratio test. To examine the shape of the associations, we also repeated the primary analysis examining associations with BMI and physical activity in deciles.

All analyses were performed using Stata version 14.1 (Stata Corporation, College Station, TX, USA), and figures were plotted in R version 3.2.3. All tests of significance were two-sided, and P values < 0.05 were considered statistically significant.

## Results

In total, 117,143 and 27,933 men were included in this analysis with valid self-reported and accelerometer-measured physical activity, respectively. Men with accelerometer-measured physical activity data were on average 56.3 years old at recruitment (standard deviation (SD) = 7.9), 97% were white, 7% were current smokers, and mean BMI was 26.7 kg/m^2^ (SD = 3.5) (Table 1). Median accelerometer-measured physical activity score was 27.4 milligravity (IQR = 10.2). In men with valid self-reported physical activity levels, median METs were 33.9 h per week (interquartile range (IQR) = 49.9) (Table 1). Men who took part in the accelerometer study were slightly more likely to have a university degree and were less likely to be current smokers than men with valid self-reported physical activity estimates (Table 1).

**Table 1:** Participant characteristics in UK Biobank male participants with valid self-reported physical activity and accelerometer data

	Accelerometer (*n* = 27,933)	Self-reported physical activity (*n* = 117,143)
Sociodemographic factors		
Age at recruitment (years), mean (SD)	56.3 (7.9)	56.2 (8.2)
White, *n* (%)	27,128 (97.4)	112,117 (96.0)
Townsend deprivation score, med (IQR)	− 2.6 (3.4)	− 2.5 (3.6)
University degree, *n* (%)	21,336 (82.5)	82,313 (79.7)
Paid employment/self-employed, *n* (%)	18,621 (66.7)	77,881 (66.5)
Current smoker, *n* (%)	1,961 (7.0)	11,172 (9.6)
Alcohol (≥ 10 g ethanol per day), *n* (%)	19,029 (68.2)	80,115 (68.5)
Health-related factors		
Vasectomy, *n* (%)	1,725 (6.2)	6,734 (5.7)
Hypertensive, *n* (%)	13,634 (48.8)	59,269 (50.6)
Family history of prostate cancer, *n* (%)	2,255 (13.6)	9,029 (13.2)
Anthropometric factors		
BMI (kg/m^2^), mean (SD)	26.7 (3.5)	27.1 (3.6)
Height (cm), mean (SD)	176.7 (6.6)	176.2 (6.7)
Waist circumference (cm), mean (SD)	93.8 (9.8)	94.7 (9.9)
Waist-to-hip ratio, mean (SD)	0.9 (0.1)	0.9 (0.1)
Body size age 10 years (plumper), *n* (%)	3,490 (12.7)	14,305 (12.4)
Height age 10 years (above average), *n* (%)	7,443 (26.9)	30,132 (26.0)
Accelerometer-measured physical activity		
Overall score (milligravity), med (IQR)	27.4 (10.2)	–
Moderate physical activity (hrs per week), med (IQR)	7.0 (6.7)	–
Light tasks (hrs per week), med (IQR)	7.4 (6.7)	–
Walking (hrs per week), med (IQR)	18.7 (9.8)	–
Sedentary (hrs per week), med (IQR)	70.8 (16.3)	–
Self-reported physical activity		
METs (hrs per week), med (IQR)	32.1 (43.7)	33.9 (49.9)
Vigorous physical activity (hrs per week), med (IQR)	1.0 (2.5)	1.0 (2.5)
Moderate physical activity (hrs per week), med (IQR)	2.0 (4.5)	2.0 (4.6)
Walking physical activity (hrs per week), med (IQR)	3.0 (5.5)	3.5 (5.3)
Leisure sedentary activity (hrs per week), mean (SD)	21.0 (21.0)	21.0 (21.0)

^a^Med (IQR) displayed where data are not normally distributed

*BMI* body mass index; *IQR* interquartile range; *med* median; *MET* metabolic equivalent of task; *SD* standard deviation

Participants with higher accelerometer measurements were on average younger, had a lower BMI, were less likely to be current smokers and more likely to be in paid employment or self-employed. These men also had higher levels of self-reported vigorous, moderate and walking physical activity, and spent less time sedentary (Table 2). Participants in the highest fifth of self-reported physical activity levels had a lower socioeconomic status, were less likely to be in paid employment or self-employed, had a higher overall accelerometer score and accelerometer-measured physical activity subtype values, and less sedentary time (Table 2).

**Table 2:** The characteristics of UK Biobank male participants by fifths of accelerometer score and overall MET hrs per week

	Accelerometer (fifths)					Overall MET hours per week (fifths)				
	Q1	Q2	Q3	Q4	Q5	Q1	Q2	Q3	Q4	Q5
Number of male participants, n	5,587	5,600	5,574	5,595	5,577	23,493	23,370	23,483	23,373	23,424
Anthropometric, sociodemographic and lifestyle										
Age at recruitment (yrs), mean (SD)	59.4 (7.2)	57.5 (7.6)	56.3 (7.8)	55.2 (7.8)	53.2 (7.7)	55.9 (7.9)	56.1 (8.1)	56.1 (8.3)	56.4 (8.3)	56.5 (8.4)
BMI (kg/m^2^), mean (SD)	27.8 (3.8)	27.1 (3.6)	26.6 (3.4)	26.4 (3.3)	25.7 (3.1)	27.6 (3.9)	27.1 (3.6)	26.9 (3.5)	26.9 (3.5)	27.0 (3.5)
Height (cm), mean (SD)	176.7 (6.7)	176.8 (6.6)	176.9 (6.5)	176.7 (6.5)	176.3 (6.6)	176.5 (6.8)	176.7 (6.7)	176.5 (6.7)	176.1 (6.7)	175.4 (6.6)
Ethnicity, *n *(% white)	5,452 (97.8)	5,462 (97.8)	5,423 (97.7)	5,416 (97.0)	5,375 (96.6)	22,313 (95.2)	22,335 (95.9)	22,499 (96.1)	22,418 (96.3)	22,552 (96.6)
Townsend, med (IQR)	− 2.6 (3.4)	− 2.7 (3.3)	− 2.7 (3.4)	− 2.6 (3.3)	− 2.5 (3.5)	− 2.6 (3.5)	− 2.5 (3.6)	− 2.6 (3.5)	− 2.5 (3.7)	− 2.2 (3.9)
Paid employment/ self-employed, *n* (%)	3,026 (54.2)	3,535 (63.1)	3,736 (67.0)	4,001 (71.5)	4,323 (77.5)	17,181 (73.1)	15,970 (68.3)	15,290 (65.1)	14,333 (61.3)	15,107 (64.5)
University degree, n (%)	4,130 (82.5)	4,293 (82.8)	4,332 (83.1)	4,329 (82.7)	4,252 (81.5)	17,278 (80.6)	17,489 (81.8)	17,407 (81.9)	16,271 (79.5)	13,868 (74.2)
Current smokers, *n* (%)	456 (8.2)	400 (7.2)	365 (6.6)	384 (6.9)	356 (6.4)	2,372 (10.1)	2,114 (9.1)	1,916 (8.2)	2,089 (9.0)	2,681 (11.5)
Alcohol consumption 10 + g ethanol per week, *n* (%)	3,611 (64.8)	3,781 (67.6)	3,843 (69.1)	3,931 (70.3)	3,863 (69.4)	15,243 (65.0)	16,181 (69.4)	16,639 (70.9)	16,247 (69.6)	15,805 (67.6)
Accelerometer-measured physical activity										
Overall score (milligravity), med (IQR)	19.0 (3.5)	23.7 (1.9)	27.4 (1.9)	31.6 (2.5)	39.2 (7.2)	24.9 (8.8)	26.7 (9.3)	27.9 (10.0)	29.0 (10.8)	30.0 (11.9)
Moderate physical activity (% time spent), med (IQR)	2.1 (2.3)	3.3 (2.7)	4.1 (3.1)	5.0 (3.6)	7.0 (4.9)	3.4 (3.4)	3.9 (3.6)	4.1 (3.7)	4.6 (4.1)	5.0 (4.6)
Light tasks (% time spent), med (IQR)	2.9 (2.7)	4.0 (3.1)	4.6 (3.6)	5.3 (4.1)	6.1 (4.9)	4.1 (3.7)	4.3 (4.0)	4.4 (3.9)	4.4 (4.0)	4.7 (4.3)
Walking (% time spent), med (IQR)	8.0 (4.1)	10.3 (4.4)	11.6 (5.1)	12.7 (5.7)	13.9 (6.9)	10.0 (5.4)	10.7 (5.4)	11.1 (5.7)	11.6 (5.9)	12.4 (6.4)
Sedentary (% time spent), med (IQR)	46.6 (9.1)	44.3 (8.6)	42.3 (8.1)	40.4 (8.3)	36.9 (9.3)	44.6 (9.4)	43.1 (9.6)	42.4 (9.1)	41.0 (9.3)	39.3 (9.9)
Self-reported physical activity										
Vigorous physical activity (hrs per week), med (IQR)	0.3 (1.5)	0.5 (2.0)	0.8 (2.2)	1.0 (2.3)	2.0 (3.2)	0.0 (0.2)	0.5 (1.0)	1.2 (1.7)	2.0 (2.8)	4.0 (5.5)
Moderate physical activity (hrs per week), med (IQR)	1.3 (3.2)	1.5 (3.5)	1.7 (4.5)	2.0 (4.3)	2.7 (5.7)	0.3 (0.7)	1.0 (1.5)	2.0 (2.3)	4.0 (4.3)	12.0 (11.0)
Walking (hrs per week), med (IQR)	2.5 (4.0)	3.0 (4.7)	3.3 (5.5)	3.5 (5.3)	3.5 (5.2)	1.0 (1.5)	2.5 (2.2)	3.5 (4.0)	5.3 (6.0)	14.0 (14.0)
Sedentary (hrs per week), med (IQR)	28.0 (21.0)	28.0 (21.0)	21.0 (21.0)	21.0 (14.0)	21.0 (14.0)	28.0 (21.0)	21.0 (21.0)	21.0 (21.0)	21.0 (21.0)	21.0 (21.0)

*BMI* body mass index; IQR interquartile range; med median; MET metabolic equivalent of task; SD standard deviation

The correlation between self-reported MET hours per week at baseline and repeat assessment (on average 4.3 years later) was relatively high (Spearman’s r = 0.61), and pairwise correlations between the biomarkers ranged from 0.85–0.55, for SHBG and free testosterone, respectively (Supplementary Table S1). Median absolute levels of self-reported physical activity and biomarkers at baseline and repeat measure are available in Supplementary Table S1. The correlations between MET hours per week and overall accelerometer score were lower at r = 0.23 and r = 0.28 in the baseline and repeat sample, respectively (mean time from baseline to accelerometer measurement = 5.7 years and mean time from repeat assessment to accelerometer measurement = 2.2 years).

The exposure–outcome associations are described below only if there was ≥ 5% difference in hormone concentrations across the range of the exposure.

### Anthropometric factors

**Fig. 1:**
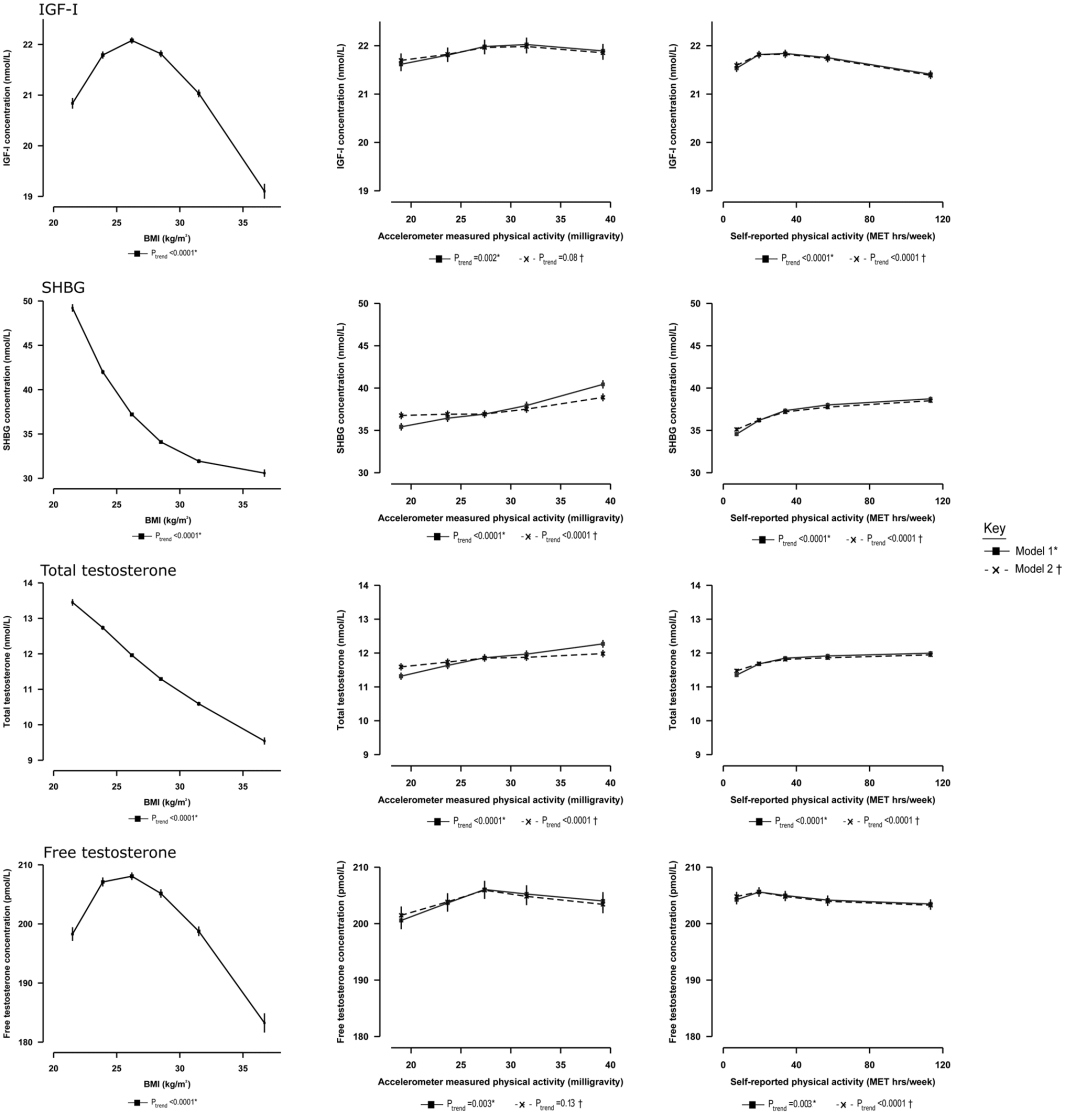
Adjusted geometric mean circulating hormone concentrations by BMI, accelerometer-measured and self-reported physical activity levels, in UK Biobank male participants. Geometric mean concentrations are presented by data points plotted as the median value within each fifth, with their 95% CIs represented as horizontal lines. P_trend_ are estimated using the analysis of variance test, with the categorical variables entered as linear values scored consecutively as the median values within each fifth. *Model 1: Estimated geometric mean concentrations adjusted for age at recruitment, geographic area, Townsend deprivation score, racial/ethnic group, height, cigarette smoking, alcohol consumption (solid line). †Model 2: Model 1 + further adjusted for BMI (dashed line). BMI = body mass index; IGF-I = insulin-like growth factor-I; SHBG = sex hormone-binding globulin

Men with BMI ≥ 35 kg/m^2^ had concentrations of SHBG, total testosterone, IGF-I and free testosterone that were 27%, 25%, 12% and 12% lower, respectively, than for men with BMI ≥ 22.5–< 25.0 kg/m^2^ (Supplementary Table S2). IGF-I and free testosterone had inverse U-shaped associations with BMI, whereas the associations with SHBG and total testosterone were approximately linear (Fig. 1). Associations with other anthropometric factors are displayed in Supplementary Table S2.

### Physical activity

#### Accelerometer-measured physical activity

Men in the highest fifth of overall accelerometer-measured physical activity had 14% and 8% higher concentrations of SHBG and total testosterone, respectively, in comparison with men in the lowest fifth (Fig. 1). After further adjustment for BMI, the magnitudes of the associations attenuated to 6% and 3%, respectively (Table 3).

**Table 3:** Adjusted geometric mean circulating hormone concentrations in UK Biobank male participants by physical activity levels with and without adjustment for BMI

	N	Med (IQR)	Model 1^a^				Model 1 + adj BMI^a^			
			IGF-I (nmol/L)	SHBG (nmol/L)	Total testosterone (nmol/L)	Free testosterone (pmol/L)	IGF-I (nmol/L)	SHBG (nmol/L)	Total testosterone (nmol/L)	Free testosterone (pmol/L)
Accelerometer-measured physical activity										
Overall										
Q1	5,567	18.99 (3.5)	21.6 (21.5–21.7)	35.4 (35.0–35.8)	11.3 (11.2–11.4)	201 (199–202)	21.7 (21.6–21.8)	36.8 (36.4–37.1)	11.6 (11.5–11.7)	201 (200–203)
Q2	5,569	23.67 (1.9)	21.8 (21.7–21.9)	36.4 (36.1–36.8)	11.6 (11.6–11.7)	204 (202–205)	21.8 (21.7–22.0)	36.9 (36.5–37.3)	11.7 (11.7–11.8)	204 (203–205)
Q3	5,545	27.37 (1.9)	22.0 (21.9–22.1)	36.9 (36.5–37.3)	11.9 (11.8–12.0)	206 (205–208)	22.0 (21.8–22.1)	36.9 (36.6–37.3)	11.8 (11.8–11.9)	206 (204–207)
Q4	5,562	31.58 (2.5)	22.0 (21.9–22.2)	37.9 (37.5–38.3)	12.0 (11.9–12.1)	205 (204–207)	22.0 (21.9–22.1)	37.5 (37.1–37.9)	11.9 (11.8–12.0)	205 (203–206)
Q5	5,541	39.24 (7.2)	21.9 (21.8–22.0)	40.4 (40.0–40.9)	12.3 (12.2–12.4)	204 (203–206)	21.9 (21.7–22.0)	38.9 (38.5–39.3)	12.0 (11.9–12.1)	203 (202–205)
P_het_			**0.0002**	** < 0.0001**	** < 0.0001**	** < 0.0001**	**0.02**	** < 0.0001**	** < 0.0001**	**0.0006**
P_trend_			**0.002**	** < 0.0001**	** < 0.0001**	**0.003**	0.08	**< 0.0001**	** < 0.0001**	0.13
Moderate physical activity (% time spent)										
Q1	5,879	1.43 (0.9)	21.8 (21.7–21.9)	36.1 (35.7–36.4)	11.6 (11.5–11.7)	204 (202–205)	21.8 (21.7–21.9)	36.8 (36.5–37.2)	11.7 (11.6–11.8)	204 (203–206)
Q2	5,376	2.86 (0.6)	21.8 (21.7–21.9)	36.3 (35.9–36.7)	11.7 (11.6–11.7)	204 (203–206)	21.8 (21.7–21.9)	36.8 (36.4–37.1)	11.7 (11.7–11.8)	204 (203–206)
Q3	5,465	4.14 (0.7)	21.9 (21.8–22.1)	36.9 (36.5–37.3)	11.8 (11.7–11.9)	205 (203–206)	21.9 (21.8–22.1)	37.1 (36.7–37.5)	11.8 (11.7–11.9)	205 (203–206)
Q4	5,587	5.86 (1.0)	22.0 (21.8–22.1)	38.3 (37.9–38.7)	12.0 (11.9–12.1)	205 (203–206)	21.9 (21.8–22.1)	38.0 (37.6–38.3)	11.9 (11.8–12.0)	204 (203–206)
Q5	5,477	9.00 (3.0)	21.9 (21.7–22.0)	39.6 (39.2–40.0)	12.1 (12.0–12.2)	202 (201–204)	21.8 (21.7–22.0)	38.4 (38.0–38.7)	11.8 (11.7–11.9)	202 (200–203)
P_het_			0.25	** < 0.0001**	** < 0.0001**	0.15	0.41	** < 0.0001**	**0.03**	0.05
P_trend_			0.28	** < 0.0001**	** < 0.0001**	0.15	0.63	** < 0.0001**	**0.02**	**0.02**
Light tasks (% time spent)										
Q1	5,685	1.29 (0.9)	21.8 (21.6–21.9)	36.7 (36.3–37.1)	11.6 (11.5–11.7)	202 (201–204)	21.8 (21.7–21.9)	37.3 (36.9–37.6)	11.7 (11.6–11.8)	203 (201–204)
Q2	5,840	3.00 (0.9)	21.8 (21.6–21.9)	37.0 (36.6–37.4)	11.7 (11.6–11.8)	203 (202–205)	21.8 (21.7–21.9)	37.5 (37.1–37.8)	11.8 (11.7–11.9)	203 (202–205)
Q3	5,345	4.43 (0.7)	21.9 (21.8–22.0)	37.0 (36.6–37.4)	11.8 (11.7–11.9)	205 (203–206)	21.9 (21.8–22.0)	37.2 (36.8–37.6)	11.8 (11.7–11.9)	205 (203–206)
Q4	5,397	6.14 (1.0)	22.0 (21.8–22.1)	37.5 (37.1–37.9)	11.9 (11.8–12.0)	205 (204–207)	21.9 (21.8–22.1)	37.2 (36.8–37.5)	11.8 (11.7–11.9)	205 (204–207)
Q5	5,517	9.14 (3.0)	21.9 (21.8–22.1)	38.9 (38.5–39.3)	12.1 (12.0–12.2)	204 (203–206)	21.9 (21.8–22.0)	37.8 (37.5–38.2)	11.9 (11.8–12.0)	204 (202–205)
P_het_			0.09	** < 0.0001**	** < 0.0001**	**0.02**	0.34	0.08	0.17	0.08
P_trend_			**0.02**	** < 0.0001**	** < 0.0001**	**0.02**	0.08	0.08	**0.02**	0.15
Walking (% time spent)										
Q1	5,636	6.29 (2.0)	21.9(21.7–22.0)	37.2 (36.8–37.6)	11.7 (11.6–11.8)	202 (201–204)	21.9 (21.8–22.1)	37.4 (37.0–37.8)	11.8 (11.7–11.9)	203 (201–204)
Q2	5,620	9.00 (1.1)	21.8(21.7–22.0)	36.9 (36.5–37.3)	11.8 (11.7–11.8)	205 (203–206)	21.8 (21.7–22.0)	36.9 (36.5–37.2)	11.8 (11.7–11.9)	205 (203–206)
Q3	5,779	11.14 (1.1)	21.8(21.7–21.9)	37.5 (37.1–37.9)	11.8 (11.8–11.9)	204 (203–206)	21.8 (21.7–21.9)	37.5 (37.2–37.9)	11.8 (11.8–11.9)	204 (203–206)
Q4	5,370	13.71 (1.3)	22.0(21.8–22.1)	37.5 (37.2–37.9)	11.8 (11.8–11.9)	204 (203–206)	21.9 (21.8–22.1)	37.5 (37.1–37.8)	11.8 (11.7–11.9)	204 (203–206)
Q5	5,379	17.57 (3.4)	21.8(21.7–22.0)	37.9 (37.5–38.3)	11.9 (11.8–12.0)	204 (203–206)	21.8 (21.7–22.0)	37.7 (37.4–38.1)	11.8 (11.7–11.9)	204 (202–205)
P_het_			0.56	**0.005**	0.07	0.19	0.43	**0.02**	0.66	0.45
P_trend_			0.79	**0.001**	**0.007**	0.19	0.55	**0.04**	0.21	0.53
Sedentary time (% time spent)										
Q1	5,667	32.71 (4.7)	21.9(21.7–22.0)	38.7 (38.3–39.1)	12.0 (11.9–12.1)	203 (201–204)	21.8 (21.7–22.0)	38.1 (37.7–38.4)	11.8 (11.7–11.9)	202 (201–204)
Q2	5,576	38.43 (2.1)	21.9(21.8–22.0)	38.3 (37.9–38.7)	12.0 (11.9–12.1)	204 (203–206)	21.9 (21.7–22.0)	37.8 (37.4–38.2)	11.8 (11.8–11.9)	204 (202–205)
Q3	5,631	42.14 (1.9)	21.9(21.7–22.0)	37.3 (36.9–37.7)	11.8 (11.7–11.9)	204 (203–205)	21.9 (21.7–22.0)	37.2 (36.9–37.6)	11.8 (11.7–11.9)	204 (202–205)
Q4	5,459	46.00 (2.0)	22.0(21.8–22.1)	36.8 (36.4–37.2)	11.8 (11.7–11.9)	205 (204–207)	22.0 (21.8–22.1)	37.0 (36.6–37.3)	11.8 (11.7–11.9)	205 (204–207)
Q5	5,451	51.14 (4.3)	21.7(21.6–21.9)	35.8 (35.4–36.2)	11.5 (11.4–11.6)	204 (202–205)	21.8 (21.7–21.9)	36.9 (36.5–37.3)	11.8 (11.7–11.9)	205 (203–206)
P_het_			0.15	** < 0.0001**	** < 0.0001**	0.23	0.45	** < 0.0001**	0.79	0.05
P_trend_			0.24	** < 0.0001**	** < 0.0001**	0.14	0.98	** < 0.0001**	0.34	**0.006**
Self-reported physical activity										
Total METs (hours per week)										
Q1	23,362	7.30 (6.3)	21.5 (21.5–21.6)	34.6 (34.4–34.7)	11.3 (11.3–11.4)	204 (204–205)	21.6 (21.5–21.7)	35.1 (34.9–35.3)	11.5 (11.4–11.5)	205 (204–206)
Q2	23,243	19.55 (6.6)	21.8 (21.8–21.9)	36.2 (36.0–36.4)	11.7 (11.6–11.7)	206 (205–206)	21.8 (21.8–21.9)	36.2 (36.0–36.4)	11.7 (11.6–11.7)	206 (205–206)
Q3	23,360	33.90 (8.7)	21.8 (21.8–21.9)	37.3 (37.1–37.5)	11.9 (11.8–11.9)	205 (204–206)	21.8 (21.8–21.9)	37.2 (37.0–37.4)	11.8 (11.8–11.9)	205 (204–206)
Q4	23,263	57.10 (16.4)	21.8 (21.7–21.8)	38.0 (37.8–38.2)	11.9 (11.9–12.0)	204 (203–205)	21.7 (21.7–21.8)	37.7 (37.6–37.9)	11.9 (11.8–11.9)	204 (203–205)
Q5	23,293	113.30 (57.2)	21.4 (21.3–21.5)	38.7 (38.5–38.9)	12.0 (12.0–12.0)	204 (203–204)	21.4 (21.3–21.5)	38.5 (38.3–38.7)	11.9 (11.9–12.0)	203 (203–204)
P_het_			** < 0.0001**	** < 0.0001**	** < 0.0001**	**0.001**	** < 0.0001**	** < 0.0001**	** < 0.0001**	**0.0002**
P_trend_			** < 0.0001**	** < 0.0001**	** < 0.0001**	**0.003**	** < 0.0001**	** < 0.0001**	** < 0.0001**	** < 0.0001**
Vigorous physical activity (hrs per week)										
Q1	34,988	0.00 (0.0)	21.5 (21.4–21.5)	35.2 (35.0–35.3)	11.4 (11.4–11.5)	204 (203–204)	21.5 (21.5–21.6)	35.6 (35.5–35.7)	11.5 (11.5–11.5)	204 (203–205)
Q2	16,703	0.33 (0.2)	21.8 (21.7–21.9)	35.9 (35.7–36.1)	11.6 (11.6–11.7)	206 (205–206)	21.8 (21.7–21.9)	35.9 (35.7–36.1)	11.6 (11.6–11.7)	206 (205–206)
Q3	23,089	1.00 (0.5)	21.7 (21.6–21.8)	37.0 (36.8–37.2)	11.8 (11.8–11.9)	206 (205–207)	21.7 (21.6–21.8)	36.9 (36.8–37.1)	11.8 (11.8–11.9)	206 (205–207)
Q4	21,102	2.25 (1.0)	21.8 (21.8–21.9)	38.3 (38.1–38.5)	12.0 (12.0–12.0)	205 (204–206)	21.8 (21.7–21.9)	38.0 (37.9–38.2)	11.9 (11.9–12.0)	205 (204–205)
Q5	20,639	5.00 (3.5)	21.6 (21.6–21.7)	39.4 (39.2–39.6)	12.1 (12.0–12.1)	203 (202–204)	21.6 (21.5–21.7)	39.0 (38.8–39.2)	12.0 (12.0–12.1)	203 (202–204)
P_het_			** < 0.0001**	** < 0.0001**	** < 0.0001**	** < 0.0001**	** < 0.0001**	** < 0.0001**	** < 0.0001**	** < 0.0001**
P_trend_			0.07	** < 0.0001**	** < 0.0001**	**0.01**	0.88	** < 0.0001**	** < 0.0001**	**0.0001**
Moderate physical activity(hrs per week)										
Q1	27,632	0.00 (0.3)	21.7 (21.6–21.7)	35.1 (35.0–35.3)	11.5 (11.4–11.5)	205 (204–205)	21.7 (21.7–21.8)	35.5 (35.3–35.6)	11.5 (11.5–11.6)	205 (204–206)
Q2	23,358	1.00 (0.5)	21.8 (21.7–21.8)	36.3 (36.1–36.5)	11.7 (11.6–11.7)	205 (204–206)	21.8 (21.7–21.8)	36.4 (36.3–36.6)	11.7 (11.6–11.7)	205 (204–206)
Q3	21,418	2.33 (0.9)	21.8 (21.7–21.9)	37.4 (37.2–37.6)	11.8 (11.8–11.9)	205 (204–206)	21.8 (21.7–21.8)	37.3 (37.1–37.5)	11.8 (11.8–11.9)	205 (204–205)
Q4	24,581	5.00 (2.0)	21.7 (21.6–21.8)	38.2 (38.0–38.4)	12.0 (11.9–12.0)	204 (204–205)	21.7 (21.6–21.7)	37.9 (37.7–38.1)	11.9 (11.9–11.9)	204 (204–205)
Q5	19,532	14.00 (8.0)	21.4 (21.3–21.4)	38.3 (38.1–38.5)	11.9 (11.9–12.0)	204 (203–204)	21.3 (21.3–21.4)	38.1 (37.9–38.3)	11.9 (11.8–11.9)	204 (203–204)
P_het_			** < 0.0001**	** < 0.0001**	** < 0.0001**	0.21	** < 0.0001**	** < 0.0001**	** < 0.0001**	**0.05**
P_trend_			** < 0.0001**	** < 0.0001**	** < 0.0001**	**0.03**	** < 0.0001**	** < 0.0001**	** < 0.0001**	**0.002**
Walks (hrs per week)										
Q1	23,725	0.67 (0.7)	21.7 (21.6–21.7)	36.0 (35.8–36.2)	11.6 (11.6–11.6)	204 (204–205)	21.7 (21.6–21.8)	36.2 (36.1–36.4)	11.7 (11.6–11.7)	205 (204–205)
Q2	25,587	2.00 (0.7)	21.8 (21.7–21.8)	36.4 (36.2–36.6)	11.7 (11.6–11.7)	205 (204–206)	21.8 (21.7–21.8)	36.5 (36.3–36.6)	11.7 (11.7–11.7)	205 (204–206)
Q3	22,998	3.50 (0.7)	21.7 (21.7–21.8)	37.2 (37.0–37.4)	11.8 (11.8–11.9)	205 (204–206)	21.7 (21.7–21.8)	37.1 (36.9–37.3)	11.8 (11.7–11.8)	205(204–205)
Q4	21,227	7.00 (1.8)	21.7 (21.6–21.8)	37.4 (37.2–37.6)	11.8 (11.8–11.9)	205 (204–205)	21.7 (21.6–21.7)	37.2 (37.0–37.4)	11.8 (11.8–11.9)	204 (204–205)
Q5	22,984	14.00 (10.5)	21.5 (21.4–21.5)	37.8 (37.6–38.0)	11.9 (11.8–11.9)	204 (203–205)	21.4 (21.4–21.5)	37.8 (37.6–38.0)	11.8 (11.8–11.9)	204 (203–205)
P_het_			** < 0.0001**	** < 0.0001**	** < 0.0001**	0.35	** < 0.0001**	** < 0.0001**	** < 0.0001**	0.21
P_trend_			** < 0.0001**	** < 0.0001**	** < 0.0001**	0.07	** < 0.0001**	** < 0.0001**	** < 0.0001**	**0.02**
Sedentary activity (hrs per week)										
Q1	38,938	14.00 (7.0)	21.6 (21.6–21.7)	38.1 (38.0–38.3)	11.9 (11.9–12.0)	204 (204–205)	21.6 (21.5–21.6)	37.1 (36.9–37.2)	11.7 (11.7–11.8)	204 (203–204)
Q2	30,781	21.00 (0.0)	21.6 (21.6–21.7)	37.1 (36.9–37.3)	11.8 (11.7–11.8)	204 (204–205)	21.6 (21.6–21.7)	37.0 (36.8–37.1)	11.7 (11.7–11.8)	204 (203–205)
Q3	27,026	28.00 (0.0)	21.6 (21.6–21.7)	36.6 (36.4–36.8)	11.7 (11.7–11.7)	204 (204–205)	21.6 (21.6–21.7)	37.0 (36.8–37.1)	11.8 (11.7–11.8)	204 (204–205)
Q4	17,684	35.00 (0.0)	21.7 (21.6–21.7)	36.5 (36.3–36.7)	11.7 (11.6–11.7)	204 (203–205)	21.7 (21.6–21.8)	37.1 (36.9–37.3)	11.8 (11.7–11.8)	204 (203–205)
Q5	22,774	49.00 (14.0)	21.5 (21.4–21.5)	35.9 (35.7–36.1)	11.6 (11.5–11.6)	204 (204–205)	21.6 (21.5–21.6)	36.9 (36.7–37.1)	11.8 (11.7–11.8)	205 (205–206)
P_het_			**0.0008**	** < 0.0001**	** < 0.0001**	0.95	**0.04**	0.59	0.09	**0.03**
P_trend_			**0.005**	** < 0.0001**	** < 0.0001**	0.83	0.70	0.28	**0.008**	**0.001**

*BMI* body mass index; *CI* confidence interval; *IGF-I* insulin-like growth factor-I; *MET *metabolic equivalent of task; *SHBG* sex hormone-binding globulin

^a^Model 1: adjusted for age at recruitment, geographic area, Townsend deprivation score, racial/ethnic group, height, cigarette smoking, alcohol consumption. Phet are assessed using the F test. Ptrend are estimated using the analysis of variance test, with the categorical variables entered as linear values scored consecutively as the median values within each fifth. P-values in bold indicate statistical significance (P<0.05)

Men in the highest fifth of time spent doing moderate and light tasks had 10% and 6% higher SHBG in comparison with men in the lowest fifth, but following adjustment for BMI, the magnitudes of the associations attenuated to 4% and 2%, respectively (Table 3). Men who spent the highest proportion of time sedentary had 8% lower SHBG, but this was attenuated to 3% lower following adjustment for BMI.

#### Self-reported physical activity

Men in the highest fifth of overall physical activity had 12% and 6% higher concentrations of SHBG and total testosterone, respectively, in comparison with the lowest fifth (Fig. 1). After further adjustment for BMI, the associations attenuated to 10% and 4%, respectively (Fig. 1).

Men in the highest fifth of vigorous, moderate and walking physical activity categories had 12%, 9% and 5% higher SHBG concentrations, respectively; and following adjustment for BMI, these associations were attenuated to 10%, 7% and 4%, respectively. Men in the highest fifth of sedentary activity had 6% lower concentrations of SHBG in comparison with men in the lowest fifth; following further adjustment for BMI, the magnitude of the association was attenuated to null (Table 3).

The associations of accelerometer-measured and self-reported physical activity with IGF-I and free testosterone were significant, but small (all < 2% differences between highest and lowest fifths).

### Further analyses

#### Percentage change in hormone concentration per 1 SD increase in exposure measures

Without adjustment for BMI, the magnitudes of the associations between accelerometer-measured physical activity and serum hormone concentrations per 1 SD increase in physical activity were generally larger than those observed with self-reported physical activity (Supplementary Table S3). Further adjustment for BMI led to attenuations of the associations with both measures of physical activity. The magnitudes of the associations with SHBG and total testosterone concentrations were generally larger for higher than for lower intensities of physical activity (whether assessed by accelerometer or self-report), both with and without adjustment for BMI. Associations in the minimally adjusted models are also displayed in Supplementary Table S3.

#### Repeat assessment and associations in tenths

When we restricted analyses to men who had attended the repeat assessment, and estimated associations using mean hormone and physical activity measurements, associations between physical activity and hormones were not materially different (Supplementary Table S4). The shapes of the associations were similar when we examined the relationship with BMI and physical activity in tenths (Supplementary Figure S2).

#### Subgroup analyses

**Fig. 2:**
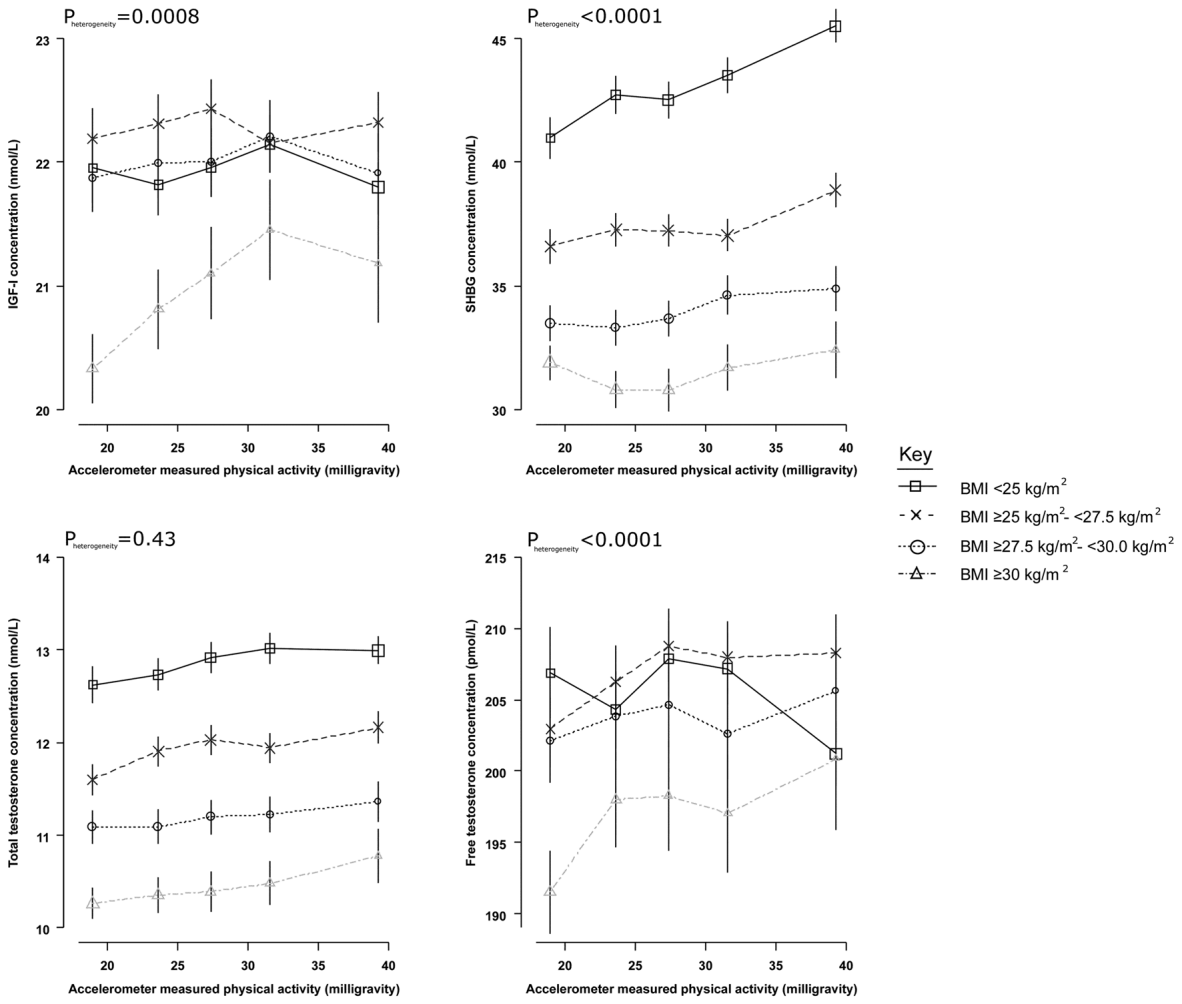
Adjusted geometric mean circulating hormone concentrations by accelerometer-measured physical activity, stratified by categories of BMI, in UK Biobank male participants. Estimated geometric mean concentrations are adjusted for age at recruitment, geographic area, Townsend deprivation score, racial/ethnic group, height, cigarette smoking, alcohol consumption. Geometric mean concentrations are presented by data points plotted as the median value within each fifth, with their 95% CIs represented as horizontal lines. P_heterogeneity_ is assessed using the F test. BMI = body mass index; IGF-I = insulin-like growth factor-I; SHBG = sex hormone-binding globulin

**Fig. 3:**
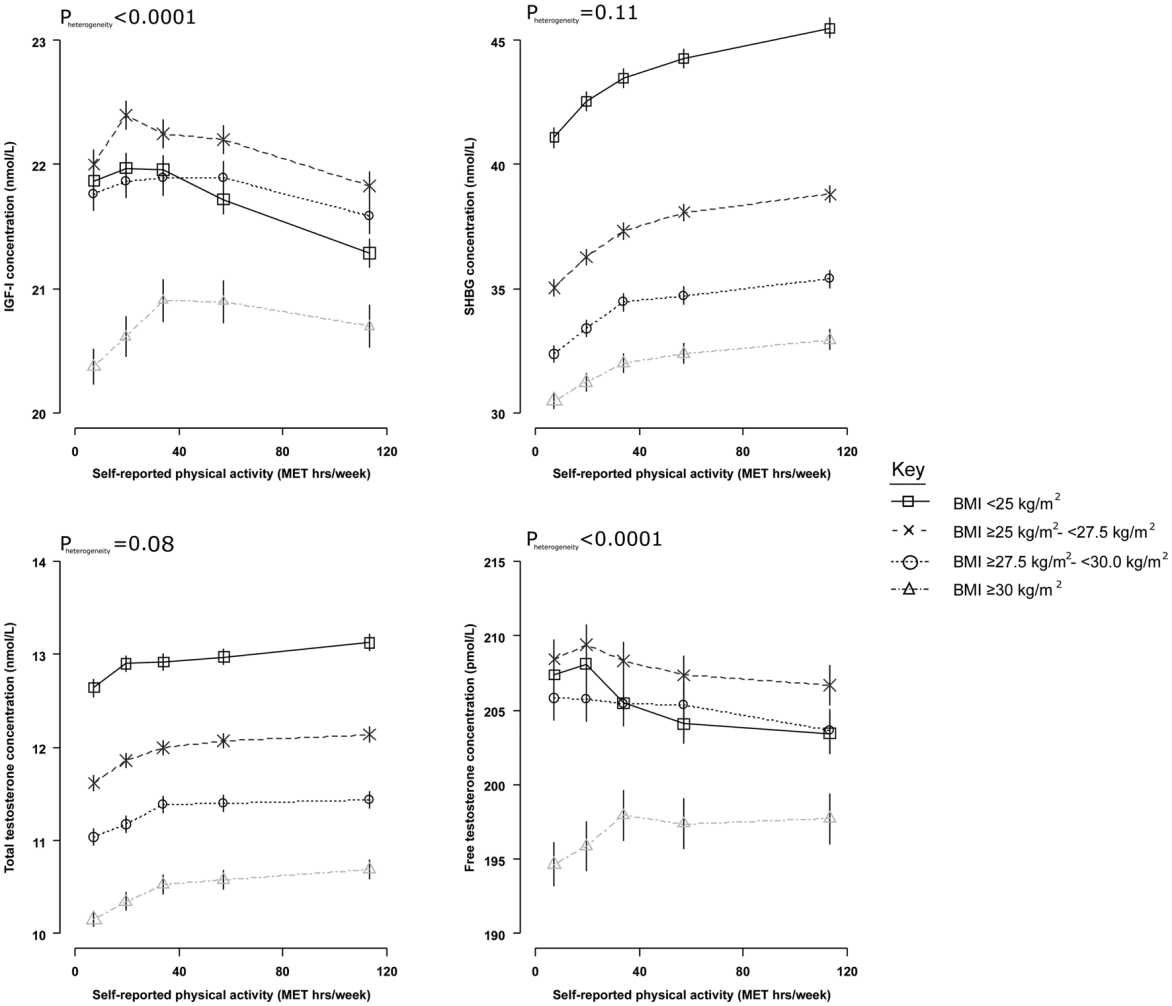
Adjusted geometric mean circulating hormone concentrations by self-reported physical activity, stratified by categories of BMI, in UK Biobank male participants. Estimated geometric mean concentrations are adjusted for age at recruitment, geographic area, Townsend deprivation score, racial/ethnic group, height, cigarette smoking, alcohol consumption. Geometric mean concentrations are presented by data points plotted as the median value within each fifth, with their 95% CIs represented as horizontal lines. P_heterogeneity_ is assessed using the F test. BMI = body mass index; IGF-I = insulin-like growth factor-I; SHBG = sex hormone-binding globulin

There was evidence of heterogeneity in the associations of both measured and self-reported physical activity with IGF-I and free testosterone by category of BMI (P_het_ < 0.01). In men with obesity, those with higher physical activity levels had higher IGF-I and free testosterone concentrations than men with lower physical activity levels, while the association of physical activity with hormones was flatter/moderately inverse in men who were not obese (Figs. 2 and 3). However, the magnitudes of the associations were small (up to 5% elevated IGF-I/free testosterone concentrations in men with obesity in the highest fifth of physical activity in comparison with the lowest). There was also evidence of heterogeneity in the association of measured physical activity and SHBG (P_het_ < 0.0001), with smaller magnitudes of associations observed in men with higher BMIs.

There was significant heterogeneity in the associations of self-reported and accelerometer-measured physical activity with SHBG and total testosterone by employment status. Men who were employed at study baseline had slightly larger magnitudes of associations than those who were not (1^st^ fifth vs the 5th) (Supplementary Table S5). Similar patterns of heterogeneity were present by physical activity at work, with slightly larger magnitudes of associations observed of physical activity with SHBG and total testosterone observed in men whose jobs did not involve manual labour, but these observed differences were modest (Supplementary Table S6).

## Discussion

This observational analysis of 117,100 men showed that those with higher physical activity levels had modestly elevated SHBG and higher total testosterone concentrations, while the differences in IGF-I and free testosterone by physical activity were very small and are unlikely to be biologically meaningful. Following adjustment for BMI, the small associations of physical activity with these biomarkers were largely attenuated.

Although there were statistically significant associations between measured, self-reported physical activity and sedentary time and IGF-I concentrations, the magnitudes of the associations were small (generally ~ 1% in the highest group in comparison with the lowest), suggesting that higher physical activity is unlikely to have a meaningful impact on cancer risk mediated by IGF-I. The magnitudes of these associations are largely consistent with other cross-sectional analyses [11, 22, 39], while results from clinical trials have been inconclusive [15–17, 40].

Physical activity may lead to elevated SHBG concentrations by reducing low-grade inflammation [41, 42], altering factors related to insulin resistance [42, 43] and possibly reducing liver fat [44]; these physiological changes may be due to modifications in body composition [45]. Our findings are largely consistent with results from clinical trials [14, 46], and some [12, 22, 47], but not all [13], cross-sectional analyses. The slightly elevated testosterone concentrations in men with higher levels of physical activity may be due to elevated SHBG concentrations, which increases the half-life of testosterone and may also result in an increase in testosterone production via the hypothalamic-pituitary–gonadal axis [48]. Findings from other studies are inconclusive [10, 12–15, 18, 46], but power was generally limited. In our analysis, the associations with physical activity were attenuated following adjustment for BMI, which may support the role of factors relating to body composition (possibly due to higher physical activity levels [45]), in driving the observed associations of physical activity with hormone concentrations.

Although there was little evidence of a biologically meaningful difference in IGF-I and free testosterone concentrations by physical activity overall, we observed evidence of a positive association among men with obesity, but not among men with a normal BMI. Furthermore, men with obesity who engaged in high levels of measured physical activity had IGF-I and free testosterone concentrations that were similar to non-obese men. Men with obesity on average have higher insulin resistance and low-grade inflammation, so these results may indicate a greater effect of physical activity on these factors [49], possibly normalising hormone concentrations [50, 51]. However, we cannot rule out bias due to better general health in these men. Clinical trials have examined possible associations between physical activity interventions and these hormones in men who are overweight and obese, but results are inconsistent (*n* < 100) [52–54]. Given the small magnitude of the associations observed in this study, larger trials may be necessary to corroborate our findings.

We also observed evidence of heterogeneity in the associations of physical activity with biomarker concentrations by employment status and manual labour at work, although the magnitudes of the associations remained small. This heterogeneity may be due to differences in the types and durations of physical activity that these groups engage in [55, 56], related to residual confounding from factors such as lifestyle differences between these populations [56] or due to chance.

The magnitudes of the associations between physical activity and hormone concentrations were slightly larger using accelerometer-measured physical activity (without adjustment for BMI). Accelerometers provide objectively measured estimates of physical activity levels and so are less likely to be biased; therefore, the greater magnitude of associations we observed might be due to reduced measurement error. Accelerometers capture general activity throughout the week, whereas self-reported METs are more likely to reflect leisure time activity [57], and consequently these physiological differences might reflect differences in the type of physical activity being captured. Associations were also larger with higher intensities of measured and self-reported physical activity, which may support the hypothesis that higher intensity of physical activity may lead to greater improvements in liver health, particularly lower liver fat [58, 59]. Therefore, our results do not preclude the possible role of more vigorous physical activity on hormone concentrations, and future research using heart rate monitors may help to elucidate this. Our findings also do not exclude the possible role of acute short-term effects or exercise regimes that may be above those observed in the general population [60].

This analysis has several strengths. It is the largest dataset currently available with hormone measurements and both objectively measured and self-reported physical activity levels, as well as physical activity intensities. The large sample size allows us to estimate moderate associations with greater precision and to detect more modest associations. The comprehensive exposure data enable us to compare associations with hormones using different methods to estimate physical activity and to account for a wide range of possible confounders and health factors. UK Biobank also collected repeat measurement data, which allowed us to verify that our results are robust over an approximately 5-year period.

A limitation of the analysis is that accelerometer-measured physical activity was recorded on average 5.7 years after blood draw, although accelerometer-measured physical activity has been shown to have good reproducibility over the medium term (2–3 years, intraclass correlation = 0.67–0.82) [61]. The UK Biobank participants are predominantly white and are healthier than the sampling population; however, the directions of the associations found here are likely to be generalizable [25, 62]. Although we have taken measures to adjust for confounding factors, residual confounding may still be present, particularly for factors which are related to health and/or body composition, which are difficult to disentangle from physical activity [45].

## Conclusion

In conclusion, the associations of physical activity with IGF-I, SHBG and testosterone were, at most, modest; and following adjustment for BMI, these associations were substantially attenuated. Therefore, it is unlikely that changes in these circulating hormones explain the associations of physical activity with risk of cancer either independently or via BMI.

## Supplementary Information

Below is the link to the electronic supplementary material.

Supplementary file1 (pdf 668 KB)

## Data Availability

UK Biobank is an open access resource, and the study website https://www.ukbiobank.ac.uk/ has information on available data and access procedures.

## Abbreviations

ANOVA: Analysis of variance
BMI: Body mass index
CI: Confidence interval
IGF-I: Insulin-like growth factor-I
IPAQ: International Physical Activity Questionnaire
IQR: Interquartile range
MET: Metabolic equivalents of task
SD: Standard deviation
SHBG: Sex hormone-binding globulin
WHR: Waist-to-hip ratio

## Code availability

Code will be made available upon request.
