# Supplementary material for: Physical activity in relation to circulating hormone concentrations in 117,100 men in UK Biobank
Source: Cancer Causes Control. 2021 Jul 3;32(11):1197–212. doi: 10.1007/s10552-021-01466-6 (PMC8492588; doi:10.1007/s10552-021-01466-6)
Supplement: Supplementary file 1 — Supplementary file1 (pdf 668 KB) [file 10552_2021_1466_MOESM1_ESM.pdf]

Physical activity in relation to circulating hormone concentrations in 117,100 men in UK Biobank–

Supplementary Tables and Figures

Table of contents

Figure S1: Participant exclusion criteria, in UK Biobank male participants…..…………...……...…….Page 2

Figure S2: Adjusted geometric mean circulating hormone concentrations by tenths of BMI, accelerometer measured and self-reported physical activity levels, in UK Biobank male participants.………………..Page 3

Table S1: Circulating biomarker and physical activity levels at baseline and repeat measurement and Spearman's pairwise correlations, in UK Biobank male participants………………………...……....Page 4

Table S2: Adjusted geometric mean circulating hormone concentrations by anthropometric factors with and without further adjustment for BMI, in UK Biobank male participants...……………………………….Page 5

Table S3: Percentage change per 1 SD increase in anthropometric and physical activity measures with and without adjustment for BMI, in UK Biobank male participants….……………………………………..Page 6

Table S4: Adjusted geometric mean circulating hormone concentrations by physical activity levels in men with two hormone measurements with and without adjustment for BMI, in UK Biobank male participants… ……………………………………………………………………………..………………….Page 7

Table S5: Adjusted geometric mean circulating hormone concentrations by physical activity level, stratified by employment status, in UK Biobank male participants ……...……………………………………….Page 8

Table S6: Adjusted geometric mean circulating hormone concentrations by physical activity, stratified by heavy manual or physical labour at work……………..…………………………………....…..…Page 9

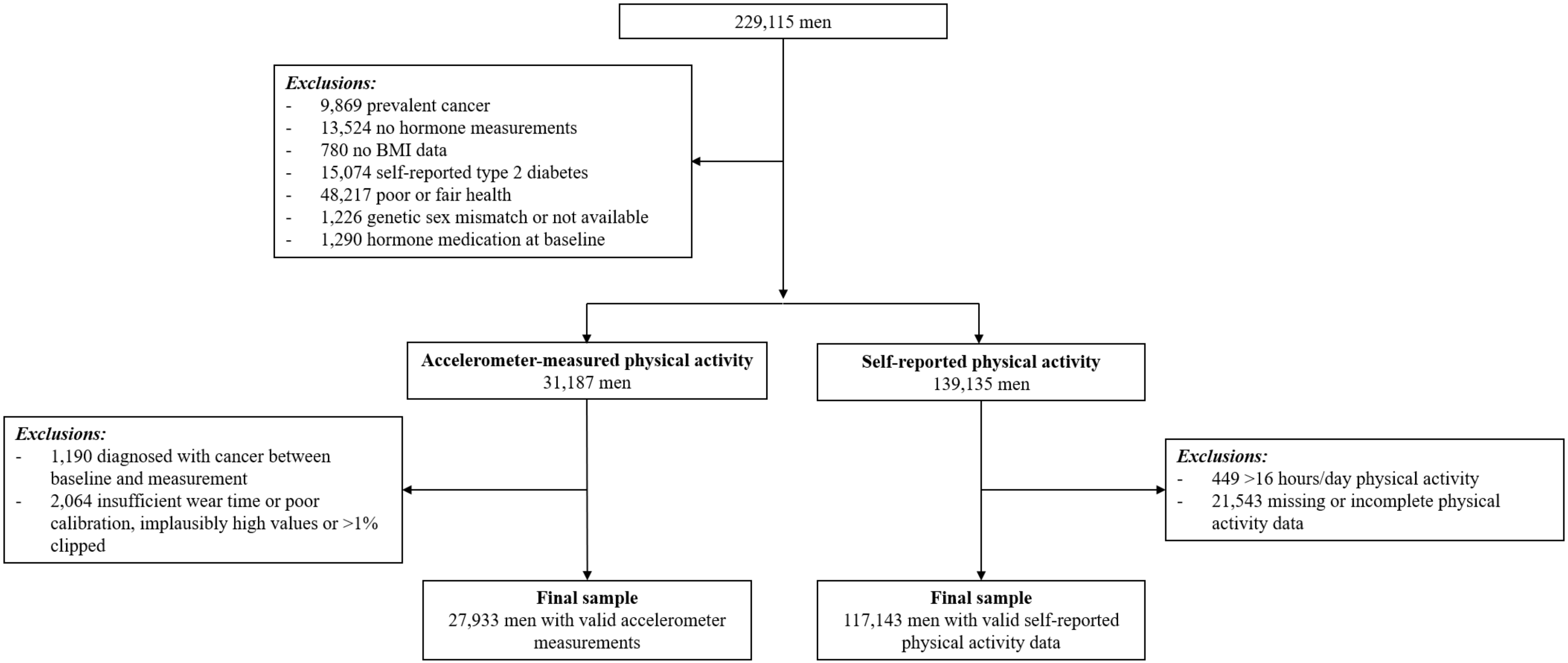

Supplementary Figure S1: **Participant exclusion criteria, in UK Biobank male participants**

Abbreviations: BMI=body mass index

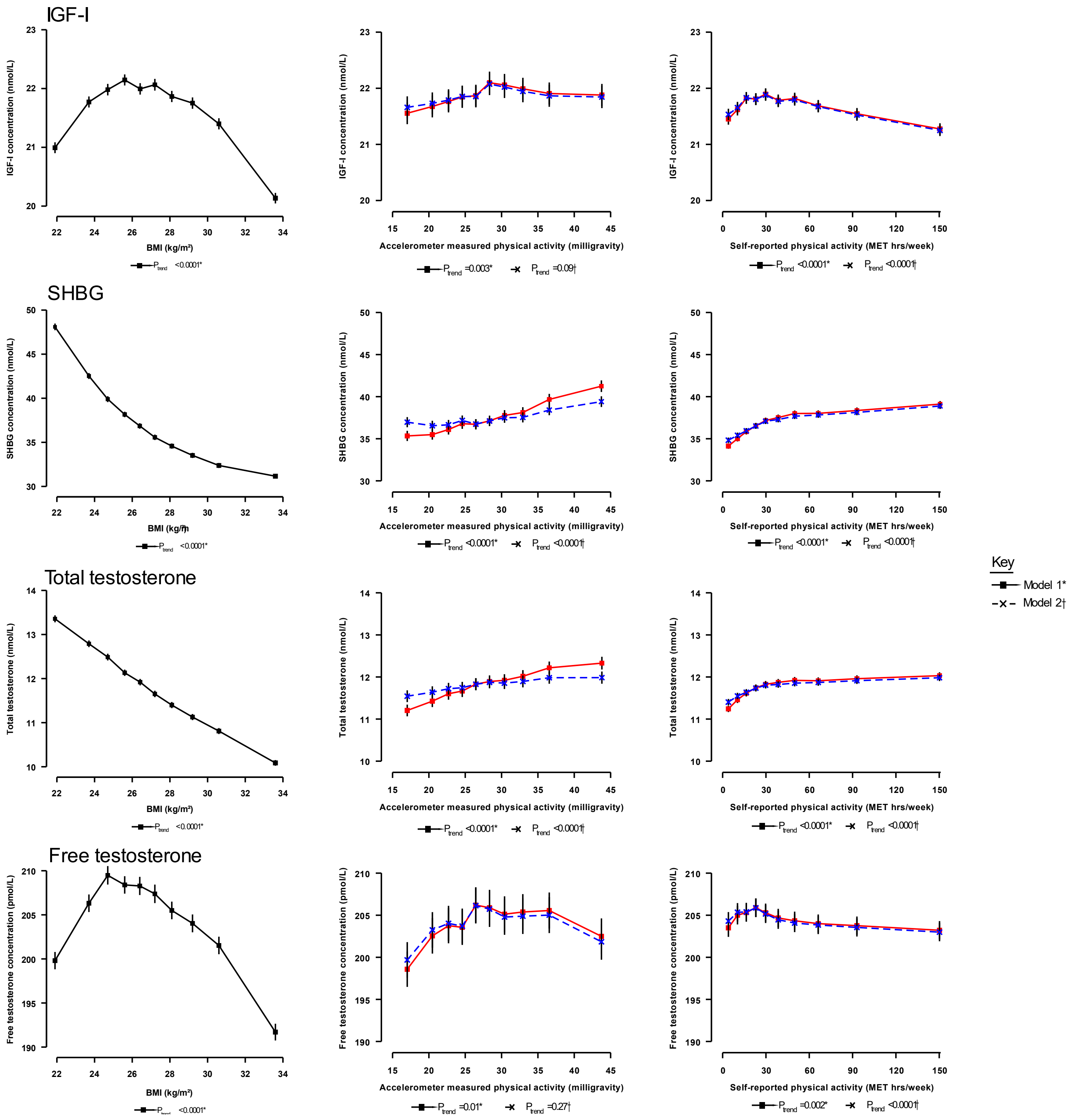

Supplementary Figure S2: **Adjusted geometric mean circulating hormone concentrations by tenths of BMI, accelerometer measured and self-reported physical activity levels, in UK Biobank male participants**

Geometric mean concentrations are presented by data points plotted as the median value within each tenth, with their 95% CIs represented as horizontal lines. P_{trend} are estimated using the analysis of variance test, with the categorical variables entered as linear values scored consecutively as the median values within each tenth.

*Model 1: Estimated geometric mean concentrations adjusted for age at recruitment, geographic area, Townsend deprivation score, racial/ethnic group, height, cigarette smoking, alcohol consumption (solid line).

† Model 2: Model 1 + further adjusted for BMI (dashed line).

Abbreviations: BMI=body mass index; IGF-I=insulin-like growth factor-I; SHBG=sex hormone-binding globulin

Supplementary Table S1: Circulating biomarker and physical activity levels at baseline and repeat measurement and Spearman's pairwise correlations, in UK Biobank male participants

		N	Correlation between baseline and repeat measurement	Median (IQR) at study baseline	Median (IQR) at repeat measure
Biomarker	IGF-I (nmol/L)	6027	0.77	22.1 (6.6)	21.6 (6.6)
	SHBG (nmol/L)	4955	0.85	38.5 (20.5)	42.4 (22.2)
	Total testosterone (nmol/L)	5965	0.66	12.0 (4.4)	12.1 (4.6)
	Free testosterone (pmol/L)	4879	0.55	207.2 (67.1)	194.1 (65.3)
Physical activity	METs (hrs per week)	5468	0.61	31.8 (45.4)	33.9 (49.9)
	Vigorous (hrs per week)	5693	0.55	1 (2.5)	1 (2.5)
	Moderate (hrs per week)	5661	0.50	2 (4.3)	2 (4.6)
	Walking (hrs per week)	5695	0.59	3 (5.5)	3.5 (5.3)

Abbreviations: IQR=interquartile range; MET=metabolic equivalent of task; IGF-I=insulin-like growth factor-I; SHBG=sex hormone-binding globulin.

Supplementary Table S2: Adjusted geometric mean circulating hormone concentrations by anthropometric factors with and without further adjustment for BMI, in UK Biobank male participants

			Model 1*				Model 1 + adj BMI*			
	N	Med (IQR)	IGF-I (nmol/L)	SHBG (nmol/L)	Total testosterone (nmol/L)	Free testosterone (pmol/L)	IGF-I (nmol/L)	SHBG (nmol/L)	Total testosterone (nmol/L)	Free testosterone (pmol/L)
BMI (kg/m^2)										
<22.5	10284	21.50 (1.4)	20.8(20.7-20.9)	49.2(48.8-49.6)	13.5(13.4-13.5)	199(197-200)	-	-	-	-
22.5-	28573	23.90 (1.2)	21.8(21.7-21.8)	42.0(41.8-42.2)	12.7(12.7-12.8)	207(207-208)	-	-	-	-
25-	41442	26.20 (1.2)	22.1(22.0-22.1)	37.2(37.1-37.3)	12.0(11.9-12.0)	208(208-209)	-	-	-	-
27.5-	31568	28.50 (1.2)	21.8(21.8-21.9)	34.1(34.0-34.2)	11.3(11.3-11.3)	205(205-206)	-	-	-	-
30-	22474	31.50 (2.1)	21.0(21.0-21.1)	31.9(31.8-32.1)	10.6(10.6-10.6)	199(198-200)	-	-	-	-
35-	4070	36.70 (2.8)	19.1(19.0-19.2)	30.6(30.2-30.9)	9.5(9.5-9.6)	183(182-185)	-	-	-	-
P_{het}			**<0.0001**	**<0.0001**	**<0.0001**	**<0.0001**				
P_{trend}			**<0.0001**	**<0.0001**	**<0.0001**	**<0.0001**				
Height (cm)										
<170	22648	167.00 (4.0)	20.9(20.9-21.0)	37.1(36.9-37.3)	11.8(11.7-11.8)	204(203-204)	20.9(20.9-21.0)	37.4(37.2-37.6)	11.8(11.8-11.9)	203(203-204)
170-	34997	172.00 (2.0)	21.4(21.4-21.5)	36.9(36.7-37.0)	11.8(11.7-11.8)	205(204-205)	21.4(21.4-21.5)	37.1(36.9-37.2)	11.8(11.8-11.8)	205(204-205)
175-	39348	177.00 (2.0)	21.7(21.6-21.7)	36.9(36.7-37.0)	11.7(11.7-11.8)	205(204-205)	21.7(21.6-21.7)	36.8(36.7-37.0)	11.7(11.7-11.8)	205(204-205)
180-	26832	182.00 (2.5)	22.0(21.9-22.0)	37.1(36.9-37.3)	11.8(11.7-11.8)	204(204-205)	22.0(21.9-22.0)	36.9(36.7-37.1)	11.7(11.7-11.8)	204(204-205)
185-	14511	187.00 (3.0)	22.2(22.1-22.3)	37.5(37.2-37.7)	11.8(11.7-11.8)	204(203-204)	22.2(22.1-22.3)	37.0(36.7-37.2)	11.7(11.6-11.7)	203(203-204)
P_{het}			**<0.0001**	**0.0002**	0.84	0.18	**<0.0001**	**<0.0001**	**<0.0001**	0.10
P_{trend}			**<0.0001**	**0.02**	0.65	0.18	**<0.0001**	**0.003**	**<0.0001**	0.08
Waist circumference (cm)										
Q1	31552	83.00 (6.0)	21.7(21.6-21.7)	44.8(44.7-45.0)	13.1(13.1-13.2)	206(205-206)	22.0(21.9-22.1)	40.8(40.5-41.0)	12.6(12.5-12.7)	208(207-209)
Q2	27376	90.00 (2.0)	22.0(22.0-22.1)	38.6(38.4-38.7)	12.2(12.2-12.3)	209(208-209)	21.9(21.8-22.0)	37.5(37.4-37.7)	12.0(12.0-12.1)	208(207-209)
Q3	28671	95.00 (2.0)	22.0(21.9-22.0)	35.8(35.6-35.9)	11.7(11.6-11.7)	207(207-208)	21.7(21.6-21.8)	36.2(36.0-36.3)	11.7(11.6-11.7)	206(205-206)
Q4	25936	100.00 (3.0)	21.6(21.6-21.7)	33.8(33.6-33.9)	11.2(11.1-11.2)	204(203-205)	21.4(21.3-21.5)	35.4(35.2-35.6)	11.4(11.3-11.4)	203(202-203)
Q5	24872	108.00 (7.5)	20.6(20.6-20.7)	31.8(31.6-31.9)	10.3(10.3-10.4)	195(194-195)	20.9(20.8-20.9)	34.7(34.5-35.0)	10.9(10.9-11.0)	196(195-197)
P_{het}			**<0.0001**	**<0.0001**	**<0.0001**	**<0.0001**	**<0.0001**	**<0.0001**	**<0.0001**	**<0.0001**
P_{trend}			**<0.0001**	**<0.0001**	**<0.0001**	**<0.0001**	**<0.0001**	**<0.0001**	**<0.0001**	**<0.0001**
Waist-to-hip ratio										
Q1	28521	0.85 (0.0)	21.8(21.7-21.8)	44.2(44.0-44.4)	12.9(12.9-13.0)	204(204-205)	21.9(21.8-21.9)	40.6(40.4-40.8)	12.3(12.3-12.4)	204(203-205)
Q2	26831	0.89 (0.0)	22.0(21.9-22.0)	38.5(38.3-38.7)	12.2(12.1-12.2)	208(207-208)	21.9(21.8-21.9)	37.5(37.3-37.7)	11.9(11.9-12.0)	206(205-207)
Q3	27928	0.92 (0.0)	21.8(21.7-21.8)	36.3(36.2-36.5)	11.7(11.7-11.8)	207(206-207)	21.7(21.6-21.7)	36.6(36.4-36.7)	11.7(11.7-11.8)	206(205-206)
Q4	27530	0.95 (0.0)	21.5(21.5-21.6)	34.5(34.3-34.6)	11.3(11.3-11.3)	204(203-205)	21.4(21.4-21.5)	35.6(35.4-35.8)	11.5(11.5-11.5)	204(203-204)
Q5	27593	1.00 (0.0)	21.0(20.9-21.0)	32.5(32.4-32.7)	10.7(10.6-10.7)	199(198-199)	21.2(21.1-21.3)	35.0(34.9-35.2)	11.3(11.2-11.3)	202(201-202)
P_{het}			**<0.0001**	**<0.0001**	**<0.0001**	**<0.0001**	**<0.0001**	**<0.0001**	**<0.0001**	**<0.0001**
P_{trend}			**<0.0001**	**<0.0001**	**<0.0001**	**<0.0001**	**<0.0001**	**<0.0001**	**<0.0001**	**<0.0001**
Childhood body size										
Thinner	46960		21.6(21.5-21.6)	36.9(36.8-37.0)	11.8(11.7-11.8)	204(204-205)	21.6(21.5-21.6)	35.7(35.6-35.8)	11.5(11.5-11.5)	204(204-205)
About average	72013		21.7(21.7-21.7)	37.2(37.1-37.3)	11.8(11.8-11.8)	205(205-205)	21.7(21.6-21.7)	37.5(37.4-37.6)	11.9(11.8-11.9)	205(204-205)
Plumper	16751		21.2(21.2-21.3)	36.5(36.3-36.7)	11.4(11.4-11.5)	200(199-201)	21.4(21.4-21.5)	38.7(38.4-38.9)	11.9(11.8-11.9)	202(201-202)
P_{het}			**<0.0001**	**<0.0001**	**<0.0001**	**<0.0001**	**<0.0001**	**<0.0001**	**<0.0001**	**<0.0001**
P_{trend}			**<0.0001**	0.27	**<0.0001**	**<0.0001**	0.97	**<0.0001**	**<0.0001**	**0.01**
Childhood height										
Shorter	26309		21.4(21.3-21.5)	37.7(37.5-37.9)	11.9(11.9-12.0)	205(204-206)	21.3(21.3-21.4)	37.0(36.8-37.2)	11.8(11.7-11.8)	205(204-205)
About average	75508		21.6(21.6-21.7)	37.0(36.9-37.2)	11.8(11.7-11.8)	204(204-205)	21.6(21.6-21.6)	37.1(37.0-37.2)	11.8(11.7-11.8)	204(204-205)
Taller	34734		21.7(21.7-21.8)	36.4(36.3-36.6)	11.6(11.5-11.6)	203(202-204)	21.8(21.7-21.8)	36.9(36.7-37.0)	11.7(11.6-11.7)	204(203-204)
P_{het}			**<0.0001**	**<0.0001**	**<0.0001**	**0.002**	**<0.0001**	0.11	**0.003**	0.12
P_{trend}			**<0.0001**	**<0.0001**	**<0.0001**	**0.001**	**<0.0001**	0.34	**0.01**	0.11

*Adjusted for age at recruitment, geographic area, Townsend deprivation score, racial/ethnic group, height, cigarette smoking, alcohol consumption. P_{het} are assessed using the F test. P_{trend} are estimated using the analysis of variance test, with the categorical variables entered as linear values scored consecutively as the median values within each fifth. P-values in bold indicate statistical significance (P<0.05).

Abbreviations: BMI=body mass index; CI=confidence interval; IGF-I=insulin-like growth factor-I; SHBG=sex hormone-binding globulin

Supplementary Table S3: Percentage change per 1 SD increase in anthropometric and physical activity measures with and without adjustment for BMI, in UK Biobank male participants

										Further adjusted for BMI							
		IGF-I (nmol/L)		SHBG (nmol/L)		Total testosterone (nmol/L)		Free testosterone (pmol/L)		IGF-I (nmol/L)		SHBG (nmol/L)		Total testosterone (nmol/L)		Free testosterone (pmol/L)	
	SD	% change	P_{trend}	% change	P_{trend}	% change	P_{trend}	% change	P_{trend}	% change	P_{trend}	% change	P_{trend}	% change	P_{trend}	% change	P_{trend}
Anthropometric factors																	
BMI (kg/m^2)	3.63																
Model 0*		-1.8%	**<0.0001**	-11.3%	**<0.0001**	-7.8%	**<0.0001**	-1.6%	**<0.0001**	-	-	-	-	-	-	-	-
Model 1†		-1.5%	**<0.0001**	-11.2%	**<0.0001**	-7.8%	**<0.0001**	-1.6%	**<0.0001**	-	-	-	-	-	-	-	-
Height (cm)	6.75																
Model 0*		2.1%	**<0.0001**	0.3%	**0.002**	-0.1%	0.18	-0.2%	**0.008**	2.1%	**<0.0001**	-0.3%	**0.003**	-0.5%	**<0.0001**	-0.3%	**0.0007**
Model 1†		2.3%	**<0.0001**	-0.3%	0.41	-0.1%	0.74	0.4%	0.18	2.2%	**<0.0001**	-0.9%	**0.008**	-0.5%	0.07	0.3%	0.27
Waist circumference (cm)	9.98																
Model 0*		-1.7%	**<0.0001**	-11.2%	**<0.0001**	-8.0%	**<0.0001**	-1.9%	**<0.0001**	-1.0%	**<0.0001**	-6.0%	**<0.0001**	-5.4%	**<0.0001**	-2.2%	**<0.0001**
Model 1†		-1.8%	**<0.0001**	-11.4%	**<0.0001**	-8.2%	**<0.0001**	-2.0%	**<0.0001**	-2.2%	**<0.0001**	-6.8%	**<0.0001**	-6.0%	**<0.0001**	-2.3%	**<0.0001**
Waist-to-hip ratio	0.06																
Model 0*		-1.8%	**<0.0001**	-10.1%	**<0.0001**	-6.5%	**<0.0001**	-1.0%	**<0.0001**	-1.4%	**<0.0001**	-4.9%	**<0.0001**	-3.0%	**<0.0001**	-0.3%	**0.0003**
Model 1†		-1.4%	**<0.0001**	-10.1%	**<0.0001**	-6.5%	**<0.0001**	-1.0%	**<0.0001**	-1.1%	**<0.0001**	-5.1%	**<0.0001**	-3.1%	**<0.0001**	-0.4%	**0.0001**
Accelerometer-measured physical activity																	
Overall accelerometer score (milligravity)	8.74																
Model 0*		0.2%	0.10	4.4%	**<0.0001**	2.4%	**<0.0001**	0.3%	0.12	0.0%	1.00	1.8%	**<0.0001**	0.8%	**<0.0001**	0.0%	0.80
Model 1†		0.4%	**0.008**	4.7%	**<0.0001**	2.6%	**<0.0001**	0.2%	0.18	0.2%	0.12	2.1%	**<0.0001**	0.9%	**<0.0001**	0.0%	0.94
Moderate physical activity (% time)	3.18																
Model 0*		-0.1%	0.43	3.4%	**<0.0001**	1.5%	**<0.0001**	-0.2%	0.18	-0.2%	0.09	1.5%	**<0.0001**	0.4%	**0.03**	-0.3%	**0.04**
Model 1†		0.0%	0.91	3.5%	**<0.0001**	1.6%	**<0.0001**	-0.2%	0.20	-0.1%	0.67	1.6%	**<0.0001**	0.4%	**0.02**	-0.3%	**0.03**
Light tasks (% time)	3.24																
Model 0*		0.3%	**0.03**	2.0%	**<0.0001**	1.4%	**<0.0001**	0.3%	0.10	0.2%	0.23	0.4%	0.06	0.4%	**0.03**	0.1%	0.40
Model 1†		0.3%	**0.01**	2.0%	**<0.0001**	1.4%	**<0.0001**	0.3%	0.12	0.3%	0.06	0.5%	**0.03**	0.4%	**0.03**	0.1%	0.47
Walking (% time)	4.48																
Model 0*		-0.1%	0.58	0.7%	**0.004**	0.5%	**0.01**	0.2%	0.15	-0.2%	0.13	0.5%	**0.04**	0.2%	0.17	0.1%	0.45
Model 1†		0.0%	0.97	0.8%	**0.0006**	0.5%	**0.004**	0.2%	0.20	-0.1%	0.36	0.5%	**0.02**	0.2%	0.15	0.1%	0.55
Sedentary time (% time)	7.26																
Model 0*		-0.2%	0.22	-2.9%	**<0.0001**	-1.3%	**<0.0001**	0.3%	0.01	0.1%	0.65	-1.2%	**<0.0001**	-0.1%	0.40	0.5%	**0.003**
Model 1†		-0.3%	**0.05**	-2.9%	**<0.0001**	-1.3%	**<0.0001**	0.3%	0.10	-0.1%	0.51	-1.2%	**<0.0001**	-0.1%	0.44	0.5%	**0.002**
Self-reported physical activity																	
METs (hrs per week)	48.44																
Model 0*		-0.9%	**<0.0001**	3.4%	**<0.0001**	1.5%	**<0.0001**	-0.3%	**0.0009**	-1.0%	**<0.0001**	3.0%	**<0.0001**	1.2%	**<0.0001**	-0.3%	**<0.0001**
Model 1†		-0.6%	**<0.0001**	3.3%	**<0.0001**	1.4%	**<0.0001**	-0.3%	**0.0006**	-0.7%	**<0.0001**	2.8%	**<0.0001**	1.1%	**<0.0001**	-0.4%	**<0.0001**
Vigorous (hrs per week)	2.85																
Model 0*		-0.3%	**<0.0001**	3.3%	**<0.0001**	1.4%	**<0.0001**	-0.3%	**0.0001**	-0.4%	**<0.0001**	2.8%	**<0.0001**	1.1%	**<0.0001**	-0.4%	**<0.0001**
Model 1†		-0.2%	**0.004**	3.2%	**<0.0001**	1.4%	**<0.0001**	-0.3%	**0.0001**	-0.3%	**<0.0001**	2.7%	**<0.0001**	1.0%	**<0.0001**	-0.4%	**<0.0001**
Moderate (hrs per week)	5.25																
Model 0*		-0.9%	**<0.0001**	2.7%	**<0.0001**	1.2%	**<0.0001**	-0.2%	**0.02**	-1.0%	**<0.0001**	2.3%	**<0.0001**	0.9%	**<0.0001**	-0.3%	**0.001**
Model 1†		-0.6%	**<0.0001**	2.6%	**<0.0001**	1.1%	**<0.0001**	-0.2%	**0.02**	-0.7%	**<0.0001**	2.1%	**<0.0001**	0.8%	**<0.0001**	-0.3%	**0.0007**
Walking (hrs per week)	5.58																
Model 0*		-0.8%	**<0.0001**	1.8%	**<0.0001**	0.8%	**<0.0001**	-0.1%	0.21	-0.9%	**<0.0001**	1.7%	**<0.0001**	0.8%	**<0.0001**	-0.1%	0.11
Model 1†		-0.5%	**<0.0001**	1.6%	**<0.0001**	0.7%	**<0.0001**	-0.1%	0.16	-0.6%	**<0.0001**	1.5%	**<0.0001**	0.6%	**<0.0001**	-0.2%	0.05
Sedentary activity (hrs per week)	14.9																
Model 0*		-0.4%	**<0.0001**	-2.1%	**<0.0001**	-1.0%	**<0.0001**	0.1%	0.13	-0.1%	0.07	-0.1%	0.16	0.3%	**0.0001**	0.4%	**<0.0001**
Model 1†		-0.2%	**0.01**	-2.2%	**<0.0001**	-1.1%	**<0.0001**	0.1%	0.26	0.0%	0.44	-0.3%	**0.006**	0.2%	**0.0109**	0.3%	**<0.0001**

P_{trend} estimated from analysis of variance model using standardised continuous variable. Bold p-values indicate statistical significance (P<0.05).
*Model 0: adjusted for age at recruitment, geographic area, Townsend deprivation score, racial/ethnic group, height, cigarette smoking, alcohol consumption.
†Model 1: Model 0 + further adjusted Townsend deprivation score, racial/ethnic group, height, cigarette smoking, alcohol consumption.

Abbreviations: BMI=body mass index; IGF-I=insulin-like growth factor-I; MET= metabolic equivalent of task; SD=standard deviation; SHBG=sex hormone-binding globulin.

Supplementary Table S4: Adjusted geometric mean circulating hormone concentrations by physical activity levels in men with two hormone measurements with and without adjustment for BMI, in UK Biobank male participants

			Baseline only		Mean baseline and repeat hormone measures	
			Model 1*	Model 2†	Model 1*	Model 2†
Hormone	Physical activity level	N	Geometric mean (95% CI)	Geometric mean (95% CI)	Geometric mean (95% CI)	Geometric mean (95% CI)
Subset with accelerometer and two hormone measurements						
IGF-I (nmol/L)	Q1	483	21.7(21.3-22.2)	21.8(21.4-22.3)	21.7(21.2-22.1)	21.7(21.3-22.2)
	Q2	462	21.7(21.3-22.2)	21.7(21.3-22.2)	21.6(21.1-22.0)	21.6(21.1-22.0)
	Q3	491	22.1(21.7-22.6)	22.0(21.6-22.5)	21.9(21.5-22.4)	21.9(21.4-22.3)
	Q4	464	21.9(21.5-22.4)	21.9(21.4-22.3)	21.7(21.2-22.1)	21.6(21.2-22.1)
	Q5	472	21.5(21.1-22.0)	21.5(21.1-22.0)	21.4(20.9-21.8)	21.4(20.9-21.8)
	P_{het}		0.48	0.59	0.51	0.59
	P_{trend}		0.65	0.42	0.36	0.28
SHBG (nmol/L)	Q1	394	36.4(35.0-37.8)	37.6(36.2-39.0)	38.1(36.7-39.5)	39.2(37.8-40.6)
	Q2	380	37.1(35.6-38.5)	37.5(36.1-38.9)	39.2(37.8-40.7)	39.6(38.2-41.0)
	Q3	403	36.5(35.1-37.9)	36.6(35.3-37.9)	38.5(37.1-39.9)	38.6(37.3-39.9)
	Q4	387	38.1(36.6-39.6)	37.6(36.3-39.1)	40.3(38.9-41.9)	39.9(38.5-41.4)
	Q5	388	41.7(40.1-43.4)	40.3(38.8-41.8)	43.6(42.0-45.2)	42.2(40.8-43.8)
	P_{het}		**<0.0001**	**0.007**	**<0.0001**	**0.007**
	P_{trend}		**<0.0001**	**0.008**	**<0.0001**	**0.003**
Total testosterone (nmol/L)	Q1	481	11.4(11.1-11.7)	11.6(11.4-12.0)	11.5(11.3-11.8)	11.7(11.5-12.0)
	Q2	458	11.4(11.1-11.7)	11.5(11.2-11.8)	11.6(11.3-11.9)	11.7(11.4-12.0)
	Q3	482	11.9(11.6-12.2)	11.9(11.6-12.2)	12.0(11.8-12.3)	12.0(11.8-12.3)
	Q4	459	11.8(11.5-12.1)	11.7(11.4-12.0)	12.0(11.7-12.3)	11.9(11.7-12.2)
	Q5	467	12.5(12.2-12.8)	12.2(11.9-12.6)	12.6(12.3-12.9)	12.4(12.1-12.7)
	P_{het}		**<0.0001**	**0.02**	**<0.0001**	**0.003**
	P_{trend}		**<0.0001**	**0.005**	**<0.0001**	**0.0003**
Free testosterone (pmol/L)	Q1	391	200(195-205)	200(195-206)	197(192-201)	197(193-202)
	Q2	373	199(194-205)	199(194-205)	196(192-200)	196(192-200)
	Q3	394	208(202-213)	207(202-213)	204(200-209)	204(200-209)
	Q4	380	205(199-210)	205(199-210)	201(196-205)	200(196-205)
	Q5	381	207(201-212)	207(201-212)	203(198-208)	203(198-208)
	P_{het}		0.10	0.14	**0.03**	0.05
	P_{trend}		**0.04**	0.06	**0.03**	**0.04**
Subset with two overall self-reported physical activity and two hormone measurements						
IGF-I (nmol/L)	Q1	973	21.6(21.3-21.9)	21.7(21.4-22.0)	21.5(21.2-21.8)	21.5(21.2-21.8)
	Q2	979	22.0(21.7-22.4)	22.1(21.7-22.4)	21.7(21.4-22.0)	21.7(21.4-22.0)
	Q3	986	21.8(21.5-22.1)	21.8(21.5-22.1)	21.8(21.5-22.1)	21.8(21.5-22.1)
	Q4	964	21.6(21.3-21.9)	21.6(21.3-21.9)	21.4(21.1-21.7)	21.4(21.1-21.7)
	Q5	961	21.3(21.0-21.6)	21.3(20.9-21.6)	21.1(20.8-21.4)	21.1(20.8-21.4)
	P_{het}		**0.02**	**0.01**	**0.01**	**0.01**
	P_{trend}		**0.008**	**0.004**	**0.006**	**0.004**
SHBG (nmol/L)	Q1	790	35.5(34.6-36.4)	36.2(35.3-37.1)	37.5(36.6-38.4)	38.1(37.2-39.0)
	Q2	815	37.3(36.3-38.3)	37.4(36.5-38.4)	39.2(38.3-40.2)	39.3(38.4-40.2)
	Q3	808	38.0(37.1-39.0)	37.8(36.9-38.7)	39.6(38.6-40.6)	39.5(38.6-40.4)
	Q4	799	38.2(37.2-39.2)	38.1(37.1-39.0)	40.8(39.8-41.9)	40.7(39.7-41.6)
	Q5	795	40.2(39.1-41.3)	39.5(38.5-40.6)	42.2(41.2-43.3)	41.7(40.7-42.7)
	P_{het}		**<0.0001**	**0.0001**	**<0.0001**	**<0.0001**
	P_{trend}		**<0.0001**	**<0.0001**	**<0.0001**	**<0.0001**
Total testosterone (nmol/L)	Q1	969	11.5(11.3-11.7)	11.6(11.4-11.8)	11.5(11.3-11.6)	11.6(11.4-11.8)
	Q2	970	11.8(11.5-12.0)	11.8(11.6-12.0)	12.1(11.9-12.3)	12.1(11.9-12.3)
	Q3	968	12.0(11.8-12.2)	11.9(11.7-12.1)	11.9(11.7-12.1)	11.8(11.7-12.0)
	Q4	956	12.0(11.8-12.2)	12.0(11.7-12.2)	12.2(12.0-12.4)	12.1(11.9-12.3)
	Q5	955	12.1(11.9-12.4)	12.0(11.8-12.3)	12.3(12.1-12.5)	12.2(12.0-12.4)
	P_{het}		**0.0001**	0.06	**<0.0001**	**<0.0001**
	P_{trend}		**0.0001**	**0.02**	**<0.0001**	**0.0001**
Free testosterone (pmol/L)	Q1	779	205(201-208)	205(202-209)	199(196-202)	199(196-202)
	Q2	805	205(201-209)	205(201-209)	205(201-208)	205(201-208)
	Q3	791	207(204-211)	207(204-211)	200(197-203)	200(197-203)
	Q4	788	205(202-209)	205(202-209)	201(197-204)	201(197-204)
	Q5	781	204(200-208)	203(200-207)	200(197-204)	200(197-203)
	P_{het}		0.81	0.77	0.12	0.14
	P_{trend}		0.54	0.38	0.71	0.55

*Model 1: adjusted for age at recruitment, geographic area, Townsend deprivation score, racial/ethnic group, height, cigarette smoking, alcohol consumption. Bold p-values indicate statistical significance (P<0.05)
†Model 2: Model 1 + further adjusted for BMI.

Abbreviations: BMI=body mass index; CI= confidence interval; IGF-I=insulin-like growth factor-I; SHBG=sex hormone-binding globulin.

Supplementary Table S5: Adjusted geometric mean circulating hormone concentrations by physical activity level, stratified by employment status, in UK Biobank male participants

		Model 1*							Model 1 + adj for BMI†		
		Employed/self-employed			Not employed				Employed/self-employed	Not employed	
Hormone	Physical activity level	N	Med (IQR)	Geometric mean (95% CI)	N	Med (IQR)	Geometric mean (95% CI)	P_{het}	Geometric mean (95% CI)	Geometric mean (95% CI)	P_{het}
Accelerometer measured physical activity											
IGF-I (nmol/L)	Q1	3013	19.19 (3.4)	22.0(21.9-22.2)	2520	18.74 (3.7)	20.8(20.6-21.0)	0.63	22.1(21.9-22.3)	20.9(20.7-21.0)	0.60
	Q2	3516	23.71 (1.9)	22.2(22.0-22.4)	2025	23.60 (1.9)	21.1(20.8-21.3)		22.2(22.0-22.4)	21.1(20.9-21.3)	
	Q3	3714	27.41 (1.8)	22.5(22.3-22.6)	1807	27.29 (2.0)	21.1(20.8-21.3)		22.5(22.3-22.6)	21.0(20.8-21.3)	
	Q4	3974	31.61 (2.5)	22.4(22.2-22.5)	1567	31.49 (2.6)	21.3(21.1-21.6)		22.4(22.2-22.5)	21.3(21.0-21.5)	
	Q5	4301	39.42 (7.5)	22.3(22.2-22.5)	1197	38.40 (6.3)	21.0(20.8-21.3)		22.3(22.1-22.4)	21.0(20.7-21.3)	
	P_{trend}			**0.02**			**0.02**		0.18	0.16	
SHBG (nmol/L)	Q1	2754	19.21 (3.3)	33.3(32.8-33.7)	2283	18.76 (3.7)	39.9(39.3-40.5)	**0.002**	34.5(34.1-35.0)	41.3(40.7-41.9)	**0.0001**
	Q2	3275	23.71 (1.9)	34.5(34.0-34.9)	1884	23.59 (1.9)	40.9(40.2-41.6)		35.0(34.6-35.5)	41.1(40.5-41.8)	
	Q3	3444	27.40 (1.9)	34.9(34.4-35.3)	1671	27.31 (1.9)	41.4(40.7-42.2)		35.0(34.6-35.4)	41.2(40.5-41.9)	
	Q4	3642	31.59 (2.5)	35.9(35.5-36.4)	1446	31.54 (2.6)	42.4(41.6-43.2)		35.7(35.3-36.1)	41.5(40.7-42.2)	
	Q5	3935	39.42 (7.4)	38.5(38.1-39.0)	1104	38.38 (6.2)	44.1(43.1-45.1)		37.1(36.7-37.6)	42.3(41.4-43.2)	
	P_{trend}			**<0.0001**			**<0.0001**		**<0.0001**	0.07	
Total testosterone (nmol/L)	Q1	3005	19.19 (3.4)	11.3(11.2-11.4)	2504	18.75 (3.7)	11.3(11.2-11.5)	0.47	11.6(11.4-11.7)	11.6(11.5-11.7)	0.23
	Q2	3505	23.71 (1.9)	11.6(11.5-11.8)	2023	23.60 (1.9)	11.6(11.5-11.8)		11.8(11.6-11.9)	11.7(11.5-11.8)	
	Q3	3709	27.40 (1.8)	11.9(11.7-12.0)	1798	27.30 (1.9)	11.8(11.7-12.0)		11.9(11.8-12.0)	11.8(11.6-12.0)	
	Q4	3961	31.60 (2.5)	12.0(11.9-12.1)	1565	31.49 (2.6)	12.0(11.8-12.1)		11.9(11.8-12.0)	11.8(11.6-11.9)	
	Q5	4282	39.41 (7.5)	12.3(12.2-12.4)	1201	38.40 (6.3)	12.1(11.9-12.4)		12.0(11.9-12.1)	11.8(11.6-12.0)	
	P_{trend}			**<0.0001**			**<0.0001**		**<0.0001**	0.06	
Free testosterone (pmol/L)	Q1	2742	19.20 (3.3)	207(205-209)	2276	18.76 (3.7)	189(187-191)	0.45	207(205-210)	190(188-192)	0.46
	Q2	3255	23.71 (1.9)	210(208-212)	1878	23.59 (1.9)	191(189-194)		210(208-212)	192(189-194)	
	Q3	3427	27.40 (1.9)	212(211-214)	1664	27.32 (1.9)	194(191-196)		212(211-214)	193(191-196)	
	Q4	3620	31.59 (2.5)	211(210-213)	1443	31.54 (2.6)	193(191-196)		211(209-213)	192(190-195)	
	Q5	3913	39.41 (7.4)	210(209-212)	1101	38.39 (6.3)	192(189-195)		210(208-212)	191(188-194)	
	P_{trend}			**0.03**			**0.03**		0.20	0.41	
Overall MET hours per week											
IGF-I (nmol/L)	Q1	17079	6.91 (6.5)	22.0(21.9-22.1)	6165	7.95 (5.9)	20.6(20.5-20.8)	0.21	22.0(22.0-22.1)	20.7(20.6-20.9)	0.28
	Q2	15877	19.55 (6.5)	22.3(22.2-22.4)	7242	19.55 (6.8)	20.9(20.8-21.0)		22.3(22.2-22.4)	20.9(20.8-21.0)	
	Q3	15213	33.85 (8.6)	22.3(22.2-22.4)	7995	34.07 (8.9)	21.0(20.9-21.1)		22.3(22.2-22.3)	21.0(20.8-21.1)	
	Q4	14261	56.87 (16.5)	22.2(22.1-22.3)	8837	57.40 (16.2)	20.9(20.8-21.0)		22.2(22.1-22.3)	20.8(20.7-20.9)	
	Q5	15032	117.30 (61.2)	21.8(21.7-21.9)	8076	107.70 (48.2)	20.6(20.5-20.7)		21.8(21.7-21.9)	20.6(20.5-20.7)	
	P_{trend}			**<0.0001**			**0.008**		**<0.0001**	**0.0003**	
SHBG (nmol/L)	Q1	15801	6.88 (6.6)	32.6(32.4-32.8)	5688	7.93 (5.9)	39.3(38.9-39.7)	**<0.0001**	33.0(32.9-33.2)	39.9(39.6-40.3)	**<0.0001**
	Q2	14577	19.55 (6.6)	34.2(34.0-34.4)	6669	19.55 (6.8)	40.7(40.3-41.0)		34.2(33.9-34.4)	40.8(40.5-41.2)	
	Q3	14041	33.85 (8.6)	35.4(35.2-35.7)	7377	33.99 (8.8)	41.4(41.1-41.8)		35.3(35.1-35.5)	41.3(41.0-41.7)	
	Q4	13105	56.76 (16.4)	36.1(35.9-36.3)	8115	57.55 (16.2)	42.1(41.8-42.5)		35.9(35.6-36.1)	41.9(41.6-42.2)	
	Q5	13772	117.30 (61.5)	36.9(36.7-37.1)	7429	107.79 (48.4)	42.8(42.4-43.2)		36.7(36.5-36.9)	42.5(42.2-42.8)	
	P_{trend}			**<0.0001**			**<0.0001**		**<0.0001**	**<0.0001**	
Total testosterone (nmol/L)	Q1	17038	6.95 (6.5)	11.4(11.3-11.4)	6136	7.93 (5.9)	11.3(11.2-11.4)	**0.04**	11.5(11.4-11.5)	11.5(11.4-11.5)	**0.004**
	Q2	15824	19.55 (6.5)	11.7(11.7-11.8)	7214	19.55 (6.8)	11.6(11.5-11.7)		11.7(11.7-11.8)	11.6(11.5-11.7)	
	Q3	15153	33.85 (8.6)	11.9(11.8-11.9)	7987	34.10 (8.9)	11.8(11.7-11.8)		11.8(11.8-11.9)	11.7(11.7-11.8)	
	Q4	14222	56.87 (16.5)	12.0(11.9-12.0)	8811	57.40 (16.1)	11.8(11.7-11.9)		11.9(11.9-12.0)	11.7(11.7-11.8)	
	Q5	14987	117.30 (61.5)	12.1(12.0-12.1)	8058	107.83 (48.2)	11.9(11.8-12.0)		12.0(12.0-12.1)	11.8(11.7-11.9)	
	P_{trend}			**<0.0001**			**<0.0001**		**<0.0001**	**<0.0001**	
Free testosterone (pmol/L)	Q1	15712	6.88 (6.6)	211(211-212)	5654	7.93 (5.9)	190(189-191)	0.24	212(211-213)	191(190-192)	0.31
	Q2	14495	19.55 (6.6)	213(212-214)	6638	19.55 (6.8)	192(190-193)		213(212-214)	192(191-193)	
	Q3	13965	33.88 (8.6)	212(211-212)	7347	34.07 (8.9)	192(191-194)		211(210-212)	192(191-193)	
	Q4	13054	56.75 (16.4)	211(210-212)	8079	57.55 (16.3)	191(190-192)		211(210-212)	190(189-191)	
	Q5	13695	117.30 (61.5)	210(209-211)	7378	107.97 (48.4)	191(189-192)		210(209-211)	190(189-191)	
	P_{trend}			**0.001**			0.49		**0.0001**	0.07	

*Model 1: adjusted for age at recruitment, geographic area, Townsend deprivation score, racial/ethnic group, height, cigarette smoking, alcohol consumption.

†Model 2: Model 1 + further adjusted for BMI.

Bold p-values indicate statistical significance ($P<0.05$)

Abbreviations: BMI=body mass index; CI= confidence interval; IGF-I=insulin-like growth factor-I; MET=metabolic equivalent of task; SHBG=sex hormone-binding globulin

Supplementary Table S6: Adjusted geometric mean circulating hormone concentrations by physical activity, stratified by heavy manual or physical labour at work, in UK Biobank male participants

		Model 1*							Model 1 + adj for BMI†		
		Job does not involve manual labour			Job involves some manual labour				Job does not involve manual labour	Job involves some manual labour	
Hormone	Physical activity level	N	Med (IQR)	Geometric mean (95% CI)	N	Med (IQR)	Geometric mean (95% CI)	P_{het}	Geometric mean (95% CI)	Geometric mean (95% CI)	P_{het}
Accelerometer measured physical activity											
IGF-I (nmol/L)	Q1	2312	19.24 (3.3)	22.2(22.0-22.4)	701	19.08 (3.4)	21.6(21.3-22.0)	0.52	22.3(22.1-22.5)	21.7(21.3-22.1)	0.49
	Q2	2655	23.68 (1.9)	22.3(22.1-22.5)	861	23.82 (1.9)	21.8(21.5-22.2)		22.3(22.1-22.5)	21.9(21.5-22.2)	
	Q3	2744	27.40 (1.9)	22.6(22.4-22.8)	969	27.41 (1.8)	22.0(21.7-22.3)		22.6(22.4-22.8)	22.0(21.7-22.3)	
	Q4	2795	31.55 (2.5)	22.6(22.4-22.7)	1178	31.73 (2.6)	22.0(21.7-22.3)		22.5(22.3-22.7)	22.0(21.7-22.2)	
	Q5	2631	39.17 (7.0)	22.5(22.4-22.7)	1670	39.94 (8.3)	21.9(21.7-22.2)		22.5(22.3-22.7)	21.9(21.7-22.2)	
	P_{trend}			**0.008**			0.31		0.08	0.51	
SHBG (nmol/L)	Q1	2108	19.24 (3.3)	32.9(32.4-33.5)	646	19.14 (3.4)	34.3(33.3-35.4)	**0.02**	34.3(33.8-34.8)	35.4(34.4-36.5)	0.24
	Q2	2472	23.68 (1.9)	34.3(33.8-34.9)	803	23.81 (1.9)	34.8(33.9-35.7)		34.9(34.4-35.4)	35.5(34.6-36.4)	
	Q3	2547	27.40 (1.9)	34.9(34.3-35.4)	897	27.41 (1.8)	34.8(33.9-35.6)		34.9(34.4-35.4)	35.2(34.4-36.1)	
	Q4	2552	31.55 (2.5)	36.0(35.5-36.6)	1089	31.68 (2.5)	35.7(34.9-36.6)		35.7(35.2-36.2)	35.8(35.0-36.5)	
	Q5	2419	39.18 (7.0)	38.7(38.1-39.3)	1516	39.88 (8.1)	38.5(37.7-39.2)		37.0(36.5-37.6)	37.2(36.5-37.9)	
	P_{trend}			**<0.0001**			**<0.0001**		**<0.0001**	**0.0004**	
Total testosterone (nmol/L)	Q1	2306	19.23 (3.3)	11.3(11.1-11.4)	699	19.07 (3.5)	11.4(11.1-11.7)	**0.03**	11.5(11.4-11.7)	11.6(11.4-11.9)	0.21
	Q2	2649	23.68 (1.9)	11.7(11.5-11.8)	856	23.82 (1.9)	11.6(11.3-11.8)		11.8(11.6-11.9)	11.7(11.5-12.0)	
	Q3	2739	27.40 (1.9)	11.9(11.7-12.0)	969	27.41 (1.8)	11.8(11.6-12.0)		11.9(11.7-12.0)	11.9(11.7-12.1)	
	Q4	2778	31.54 (2.5)	12.1(11.9-12.2)	1182	31.73 (2.6)	11.8(11.6-12.0)		12.0(11.9-12.1)	11.8(11.6-12.0)	
	Q5	2622	39.17 (7.0)	12.4(12.2-12.5)	1660	39.88 (8.3)	12.2(12.0-12.4)		12.1(11.9-12.2)	12.0(11.8-12.2)	
	P_{trend}			**<0.0001**			**<0.0001**		**<0.0001**	**0.02**	
Free testosterone (pmol/L)	Q1	2099	19.24 (3.3)	207(204-209)	643	19.14 (3.4)	206(202-211)	0.49	208(205-210)	207(202-211)	0.47
	Q2	2459	23.68 (1.9)	211(209-213)	796	23.82 (1.9)	208(204-212)		211(209-213)	208(204-212)	
	Q3	2535	27.40 (1.9)	212(210-215)	892	27.41 (1.8)	213(209-216)		212(210-215)	212(209-216)	
	Q4	2536	31.55 (2.5)	213(211-215)	1083	31.69 (2.5)	209(205-212)		212(210-214)	209(205-212)	
	Q5	2408	39.17 (7.0)	211(209-213)	1505	39.88 (8.2)	209(206-212)		211(209-213)	209(206-212)	
	P_{trend}			**0.01**			0.49		0.12	0.73	
Overall MET hours per week											
IGF-I (nmol/L)	Q1	14410	6.83 (6.6)	22.1(22.0-22.2)	2657	7.53 (6.3)	21.7(21.5-21.9)	**<0.0001**	22.2(22.1-22.3)	21.7(21.5-21.9)	**<0.0001**
	Q2	12524	19.42 (6.6)	22.4(22.3-22.5)	3350	19.80 (6.5)	22.0(21.8-22.1)		22.4(22.3-22.5)	22.0(21.8-22.2)	
	Q3	11225	33.75 (8.6)	22.5(22.4-22.6)	3981	34.47 (9.0)	21.8(21.7-22.0)		22.4(22.3-22.5)	21.8(21.7-22.0)	
	Q4	8647	55.10 (14.9)	22.4(22.3-22.5)	5607	59.80 (17.2)	21.9(21.7-22.0)		22.4(22.3-22.5)	21.9(21.7-22.0)	
	Q5	3657	98.21 (33.4)	22.3(22.1-22.4)	11372	127.17 (66.8)	21.5(21.4-21.6)		22.2(22.1-22.4)	21.5(21.4-21.6)	
	P_{trend}			0.06			**<0.0001**		0.43	**<0.0001**	
SHBG (nmol/L)	Q1	13332	6.83 (6.7)	32.5(32.3-32.7)	2457	7.50 (6.3)	33.0(32.5-33.5)	**<0.0001**	33.0(32.8-33.2)	33.8(33.3-34.2)	**<0.0001**
	Q2	11488	19.48 (6.6)	34.2(33.9-34.4)	3087	19.80 (6.5)	34.1(33.6-34.6)		34.2(33.9-34.4)	34.3(33.9-34.8)	
	Q3	10316	33.77 (8.7)	35.6(35.4-35.9)	3719	34.47 (8.9)	34.8(34.4-35.3)		35.4(35.2-35.7)	35.0(34.6-35.4)	
	Q4	7928	55.10 (14.9)	36.4(36.1-36.7)	5172	59.60 (17.0)	35.7(35.3-36.0)		36.0(35.7-36.3)	35.6(35.3-36.0)	
	Q5	3334	98.20 (33.2)	37.3(36.8-37.7)	10435	127.30 (66.9)	36.8(36.5-37.1)		36.8(36.3-37.2)	36.5(36.3-36.8)	
	P_{trend}			**<0.0001**			**<0.0001**		**<0.0001**	**<0.0001**	
Total testosterone (nmol/L)	Q1	14383	6.83 (6.6)	11.4(11.3-11.4)	2643	7.53 (6.3)	11.4(11.3-11.5)	**<0.0001**	11.5(11.4-11.5)	11.6(11.4-11.7)	**<0.0001**
	Q2	12467	19.46 (6.6)	11.7(11.7-11.8)	3354	19.80 (6.5)	11.7(11.6-11.8)		11.7(11.7-11.8)	11.7(11.6-11.8)	
	Q3	11171	33.75 (8.6)	11.9(11.9-12.0)	3975	34.50 (9.0)	11.8(11.7-11.9)		11.9(11.8-11.9)	11.8(11.7-11.9)	
	Q4	8624	55.10 (14.9)	12.1(12.0-12.2)	5591	59.73 (17.3)	11.8(11.7-11.9)		12.0(11.9-12.1)	11.8(11.7-11.9)	
	Q5	3652	98.22 (33.4)	12.2(12.1-12.3)	11332	127.10 (66.9)	12.0(11.9-12.1)		12.1(12.0-12.2)	12.0(11.9-12.0)	
	P_{trend}			**<0.0001**			**<0.0001**		**<0.0001**	**<0.0001**	
Free testosterone (pmol/L)	Q1	13255	6.83 (6.7)	211(210-212)	2445	7.50 (6.3)	211(209-213)	0.15	212(211-213)	212(210-214)	0.15
	Q2	11419	19.48 (6.6)	213(212-214)	3074	19.80 (6.5)	212(210-214)		213(212-214)	213(211-215)	
	Q3	10259	33.77 (8.7)	212(211-213)	3700	34.48 (8.9)	211(210-213)		211(210-212)	211(210-213)	
	Q4	7900	55.10 (14.9)	212(211-213)	5149	59.60 (17.1)	210(209-212)		212(211-213)	210(209-212)	
	Q5	3326	98.20 (33.2)	212(210-213)	10366	127.30 (66.6)	210(209-211)		211(209-213)	210(208-211)	
	P_{trend}			0.91			**0.03**		0.39	**0.005**	

*Model 1: adjusted for age at recruitment, geographic area, Townsend deprivation score, racial/ethnic group, height, cigarette smoking, alcohol consumption.

†Model 2: Model 1 + further adjusted for BMI.

Bold p-values indicate statistical significance (P<0.05)

Abbreviations: BMI=body mass index; CI= confidence interval; IGF-I=insulin-like growth factor-I; MET=metabolic equivalent of task; SHBG=sex hormone-binding globulin
